# Recent Epidemiological Trends of Dengue in the French Territories of the Americas (2000–2012): A Systematic Literature Review

**DOI:** 10.1371/journal.pntd.0003235

**Published:** 2014-11-06

**Authors:** Maïna L'Azou, Anne-Frieda Taurel, Claude Flamand, Philippe Quénel

**Affiliations:** 1 Global Epidemiology Department, Sanofi Pasteur, Lyon, France; 2 Institut Pasteur in French Guiana, Cayenne, French Guiana; University of Heidelberg, Germany

## Abstract

Dengue is a public health concern across the globe, and an escalating problem in the Americas. As part of a wider programme (covering Latin America and South East Asia) to characterize the epidemiology of dengue in dengue endemic areas, we undertook a systematic literature review to assess epidemiological trends (incidence, timing and duration of outbreaks/epidemics, age and sex distribution, serotype distribution, seroprevalence and disease severity) for dengue across the French Territories of the Americas (FTA), in French Guiana, Guadeloupe, Martinique, Saint Martin and Saint Barthélemy between 2000 and 2012 (CRD42012002341: http://www.crd.york.ac.uk/prospero/display_record.asp?ID=CRD42012002341). Of 413 relevant data sources identified, 45 were eligible for inclusion. A large proportion of the available data were from national surveillance reports, and 12 publications were from peer-reviewed journals. During the review period, 3–5 epidemics were identified in each of the island territories and French Guiana, and epidemics were often associated with a shift in the predominant circulating dengue virus serotype. Substantial gaps in epidemiological knowledge were identified. In particular, information regarding dengue virus genotype distribution, seroprevalence and age distribution of dengue were lacking. Additionally, much of the available data were from epidemic years; data from inter-epidemic periods were sparse. Nevertheless, the available epidemiological data showed that dengue is endemic across the FTA and suggest an evolution towards hyperendemicity, highlighting the need to continue the efforts with the existing surveillance programmes to assist in planning an effective vaccination programme once a dengue vaccine is deployed.

**Protocol registration:**

PROSPERO CRD42012002341

## Author Summary

Dengue disease is a mosquito-borne viral illness and is a major health concern in the Americas. We conducted a literature analysis and review to describe the epidemiology of dengue in the French Territories of the Americas (FTA), comprising French Guiana, Guadeloupe, Martinique, Saint Martin and Saint Barthélemy, to determine the impact of dengue on the population and to identify research priorities. We used well-defined methods to search for and identify relevant research conducted between 2000 and 2012. We identified an increase in the frequency, magnitude and severity of dengue epidemics across all the territories, an increase in the co-circulation of serotypes and the evolution to a hyperendemic state. Countries sharing geographical and environmental characteristics (e.g., Martinique and Guadeloupe, and Saint Barthélemy and Saint Martin) also shared characteristics in dengue epidemiology and its evolution, including timing of epidemics and circulating serotypes. Gaps in epidemiological knowledge provide several avenues for research, such as dengue virus genotype distribution, seroprevalence and age distribution of dengue cases. Epidemiological and virological surveillance of dengue in the FTA is evolving, and improved knowledge of the disease in these territories will improve anticipation of epidemics and aid implementation of control measures.

## Introduction

**Figure 1:**
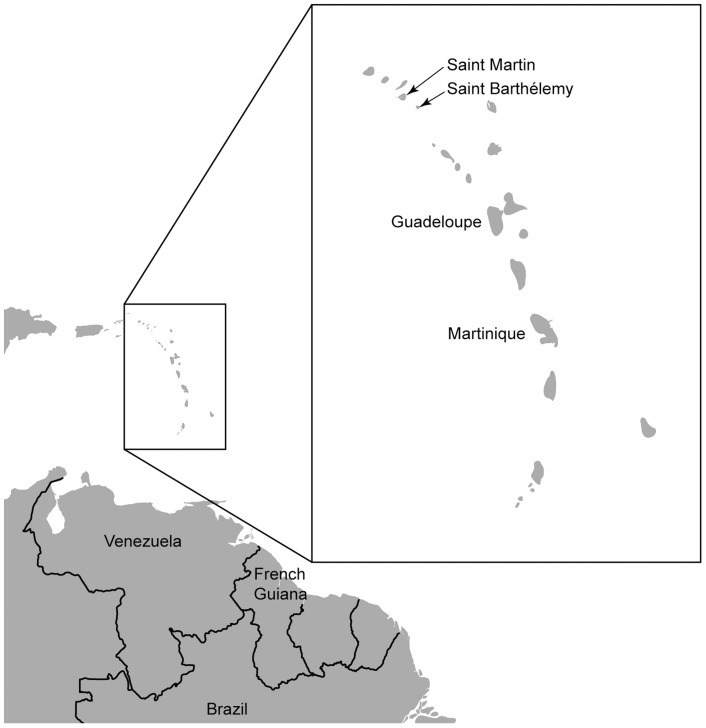
Map showing the location of the French Territories of the Americas French Guiana, Guadeloupe, Martinique, Saint Martin and Saint Barthélemy.

Dengue is caused by the dengue virus (DENV), of which there are four serotypes (DENV-1–4) that are transmitted to humans by infected *Aedes sp.* mosquitoes. Infection produces a spectrum of illness, ranging from inapparent or mild, non-specific febrile syndrome to classic dengue fever (DF), or to severe disease forms including dengue haemorrhagic fever (DHF) and dengue shock syndrome (DSS). Dengue is a public health concern across the globe [1] and an escalating problem in the Americas, where reported cases have increased from 1 million during the 1980s to 4.7 million during 2000–2007 [2]. This review describes the epidemiology of dengue in the French Territories of the Americas (FTA), comprising French Guiana, Martinique, Guadeloupe, Saint Martin and Saint Barthélemy (Figure 1), for the period of 2000–2012.

The geographical features of the FTA vary considerably with regards to the demographic profile and climate, which make comparisons across the territories difficult. However, the territory pairings of Martinique and Guadeloupe, and Saint Martin and Saint Barthélemy share some geographical characteristics, making it easier to compare the epidemiology of dengue between these two sets of territories. Martinique and Guadeloupe have tropical climates, with rainy season from July–October in Martinique [3] and May–October in Guadeloupe [4]. Saint Martin and Saint Barthélemy also have tropical climates, but these are drier than those of Martinique and Guadeloupe, with a dry season during December–May, with punctual shortages of water, and a rainy season from July–October [4]. By contrast, French Guiana has an equatorial climate, with two rainy seasons (April–July and December–March) and a dry season from August to November [5], [6].

### History of dengue in the Americas

The first epidemic of DF in French Guiana was identified in 1943 [7]. During the 1950s and 1960s, an eradication programme was conducted across the Americas to eradicate *Ae. Aegypti (Linnaeus)*, which is also the principal vector mosquito for the yellow fever virus. *Ae. aegypti* was eradicated from French Guiana in 1958 [8]. However, when the eradication programme was stopped, the mosquito re-infested the countries from which it had been eliminated. The mosquito was newly identified in French Guiana in 1964, and the first serological confirmation of DENV in this region was during an outbreak of DF in 1965 [8]. DHF was first reported in the Americas in 1981 during an epidemic in Cuba; since this time DHF and DSS have been frequently reported in the Americas and the Caribbean [9]. During the 1980s and 1990s, dengue spread throughout the Americas and Caribbean. DENV-2 was first isolated in French Guiana in 1970, in Saint Martin in 1985 and in Martinique and Guadeloupe in 1989 [8]. DENV-1 was reported in Martinique and Saint Martin in 1977 and in French Guiana in 1978 [8], [10], [11]. In Saint Martin and Saint Barthélemy the first reports of DENV-4 occurred in 1981 [12] and in French Guiana, Martinique and Guadeloupe in 1982 [8]. DENV-3 re-emerged in French Guiana, Martinique, Guadeloupe and Saint Martin in 1999 [8]. All four DENV serotypes have been circulating in coastal French Guiana since the first DHF cases were reported in 1992 [5].

### Dengue surveillance systems in the French Territories of the Americas

Active and passive surveillance systems coordinated by the Regional office of the French National Institute for Public Health Surveillance (Cire AG) detect and monitor dengue outbreaks across the FTA. Epidemiological surveillance is based on three categories: clinically suggestive cases (dengue-like syndromes), laboratory-confirmed cases and hospitalized cases. Data are provided by sentinel networks of general practitioners (GPs), medical biology laboratories, public hospitals and, in French Guiana, health centres [13]. Surveillance of clinically suggestive dengue cases is based on the weekly reporting of dengue-like syndromes by GPs and health centres. The Cire AG collect weekly the number of dengue-like syndromes diagnosed the preceding week [14], and the total number of cases is extrapolated to the whole territory [11]. Surveillance of laboratory-confirmed dengue cases is based on the weekly follow-up of laboratory-confirmed dengue cases (positive NS1 antigen detection and/or positive anti-DENV immunoglobulin M [IgM] detection and/or reverse transcription-polymerase chain reaction and/or viral isolation) performed by hospitals and private laboratories. Samples are sent by GPs from the sentinel network during inter-epidemic periods and by hospitals during epidemic periods. The information is then collated in a computerized database by the National Reference Center of arboviruses of the Institut Pasteur in French Guiana (CNR) [15]. Virological surveillance of circulating serotypes is performed by the CNR and the hospital of Fort-de-France in Martinique [16]. In Martinique, French Guiana and Guadeloupe, suspected dengue deaths are submitted to clinical and biological experts and infectious disease specialists to determine whether there is a link with DENV infection [13], [17].

The Programme de Surveillance, d'Alerte et de Gestion des épidémies de dengue (PSAGE) is a programme for the surveillance and control of dengue epidemics across the FTA [18] based on the integrated management strategy recommended by the Pan American Health Organization (PAHO) [19], [20]. PSAGE declares an epidemic if the number of dengue-like syndrome cases exceeds the maximum expected value for 5 consecutive weeks and if the number of biologically confirmed cases exceeds the maximum expected value for 4 consecutive weeks. The end of an epidemic is declared when the estimated number of dengue-like syndrome cases and biologically confirmed cases fall below the maximum expected values for 2 consecutive weeks.

The case definitions for dengue and dengue severity used in the FTA have varied over time. Before 2010, the World Health Organization (WHO) 1997 case definition was used [21]. A local case definition was developed in 1998 (Institut de Veille Sanitaire [InVS] 98), which took into account the non-haemorrhagic, visceral severe forms of dengue observed in the FTA. Since 2010, the 2009 WHO case has been used in addition to the InVS 98 case definition [22].

As part of a wider programme (covering Latin America and South East Asia) [23] to characterize the epidemiology of dengue in dengue endemic areas, a systematic literature review was conducted to describe the epidemiology of dengue reported in the FTA between 1 January 2000 and 29 March 2012. The objectives of this literature review were to describe the epidemiology of dengue, in terms of incidence, serotype distribution, age and sex distribution, and seroprevalence, in French Guiana, Martinique, Guadeloupe, Saint Martin and Saint Barthélemy and to identify gaps in epidemiological knowledge requiring further research.

## Methods

A Literature Review Group developed the protocol for conducting this literature review based on the preferred reporting items of systematic reviews and meta-analyses (PRISMA) guidelines [24]. The protocol was registered on PROSPERO, an international database of prospectively registered systematic reviews in health and social care managed by the Centre for Reviews and Dissemination, University of York (CRD42012002341: http://www.crd.york.ac.uk/prospero/display_record.asp?ID=CRD42012002341) on 30 April 2012. The Literature Review Group was actively involved in defining the inclusion/exclusion criteria and guided the search and selection process described below. Relevant articles were expected to be heterogeneous with respect to data selection, numbers and classification of cases. As combining methodologically incomparable studies would have serious implications for the validity and generalizability of findings, a meta-analysis was not conducted.

Given the 3–5-year periodicity of dengue outbreaks [25], we estimated that a time period of at least 10 years would be required to accurately reflect the recent changes in dengue epidemiology. Additionally, a >10-year period would allow observation of serotype distribution over time and through several epidemics. For convenience, we chose to start our review period on 1 January 2000 and set the cut-off as 29 March 2012, the date when we initiated this review. Furthermore, we hypothesised that setting the start date as 1 January 2000, as opposed to an earlier date, would limit the bias that any differences in surveillance practices over time would have on the results.

### Search strategy and selection criteria

Specific search strings were devised for each database to be searched, with reference to the expanded Medical Subject Headings thesaurus, broadly encompassing the terms ‘dengue’, ‘epidemiology’ and ’Martinique and/or Guadeloupe and/or Saint Martin and/or Saint Barthélemy and/or French Guiana’. Search terms were tailored for each database with the aim of increasing the query's sensitivity and specificity.

Only studies published in French or English between 1 January 2000 and 29 March 2012 were included. For databases that did not allow language and/or date limitations, references not meeting these criteria were deleted manually at the first review stage. No limits by sex, age or ethnicity of study participants or by study type were imposed. Single-case reports (except reports of deaths), incomplete surveillance reports and studies that only reported data for the period before 1 January 2000 were excluded. As duplicate publication of data (e.g., in meta-analyses and other reviews) could lead to oversampling and overestimates of the incidence of dengue, editorials involving previously published peer-reviewed data were also excluded. Additional publications and unpublished data sources meeting the inclusion criteria were eligible if recommended by a consensus of the Literature Review Group.

Between 29 March 2012 and 11 April 2012, we searched nine databases, 14 on-line sources that included Institute Pasteur and surveillance system websites and grey literature (Table S1) for epidemiological studies of dengue in the FTA published between 1 January 2000 and 29 March 2012. Literature from key infectious disease, tropical medicine and paediatric conferences were also searched.

Duplicate citations were removed. The Literature Review Group first reviewed the titles and abstracts of the identified articles and a further selection was made based on review of the full text in accordance with the review objectives. We chose not to exclude articles and other data sources or formally rank them on the basis of the quality of evidence. Although we recognize that assessment of study quality can add value to a literature review, the consensus of the Literature Review Group was that given that a high proportion of available data were expected to come from surveillance data, such quality assessment would not add value in this case due to the nature of surveillance data (passive reporting of clinically suspected dengue). As our primary objective was to describe the recent evolution of dengue, rather than to quantify disease in absolute terms, we therefore retained all available data sources.

Data from the selected sources were collated and summarized using a data extraction instrument developed as a series of Excel (Microsoft Corp., Redmond, WA, USA) spreadsheets. Data from literature reviews of previously published peer-reviewed studies and pre-2000 data published within the search period were not extracted. Incidence rates were calculated using the number of cases from the selected publications and the population of the associated year issued by the Institut National de la Statistique et des Études Économiques (http://insee.fr/fr/default.asp), with the exception of the incidence rates in Saint Martin and Saint Barthélemy prior to 2006, for which population data were not available. For these territories, incidence rates prior to 2006 were estimated based on the 2006 population. We refer to outbreaks of dengue as epidemics if the epidemic was confirmed by PSAGE [26]. The original data sources and the extraction tables were made available to all members of the Literature Review Group for review and analysis.

## Results

**Figure 2:**
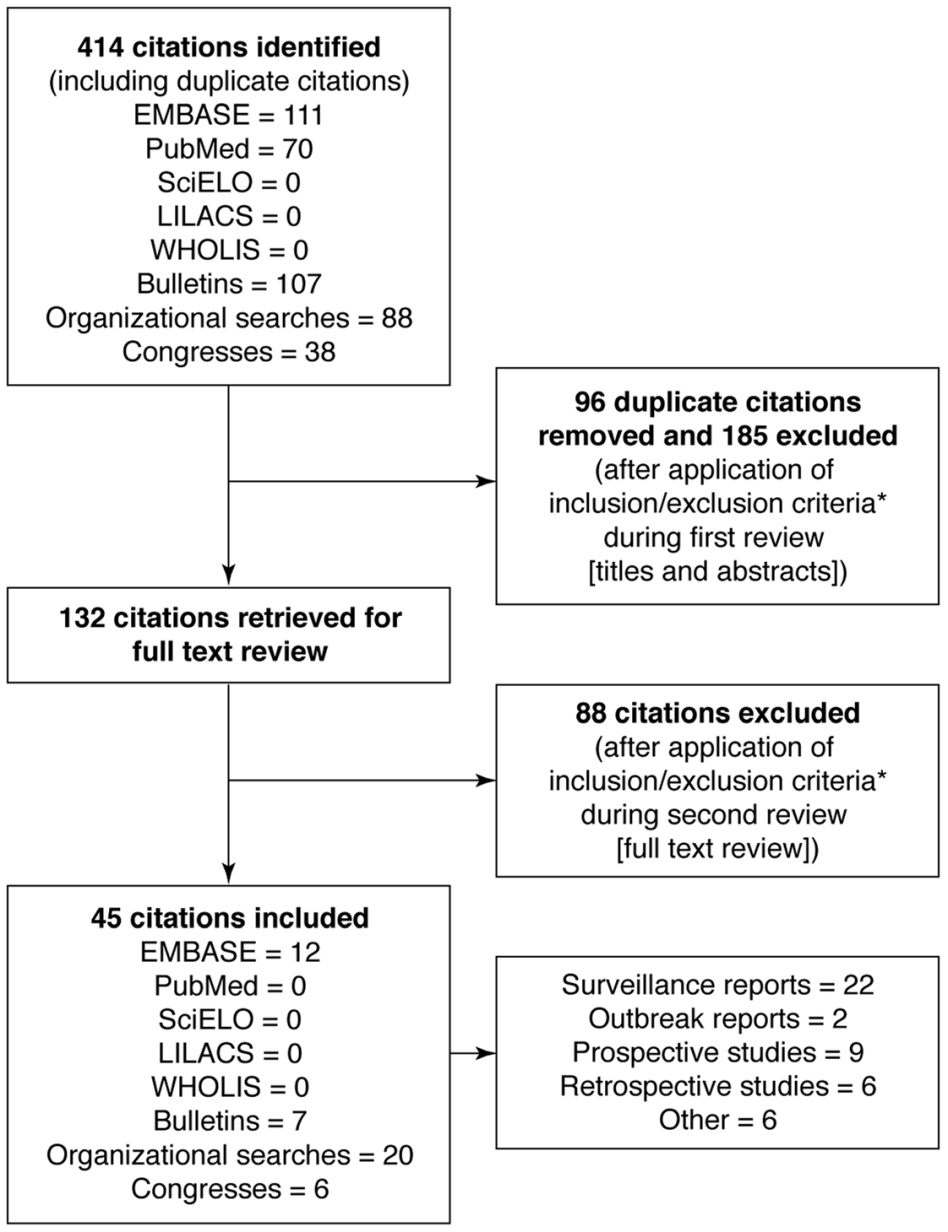
Results of the literature search and evaluation of identified studies according to PRISMA. EMBASE, Excerpta Medica Database; LILACS, Latin American and Caribbean Health Science Literature; PRISMA, preferred reporting items of systematic reviews and meta-analyses; WHOLIS, World Health Organization Library database; SciELO, Scientific Electronic Library Online. All references identified in the on-line database searches were assigned a unique identification number. After a review of the titles and abstracts duplicates and articles were removed according to the following inclusion/exclusion criteria. Studies published in French or English between 1 January 2000 and 29 March 2012 were included. No limits by sex, age or ethnicity of study participants or by study type were imposed. Single-case reports (except reports of deaths), incomplete surveillance reports and studies that only reported data for the period before 1 January 2000 were excluded. As duplicate publication of data (e.g., in meta-analyses and other reviews) could lead to oversampling and overestimates of the incidence of dengue, editorials involving previously published peer-reviewed data were also excluded. A further selection was made based on review of the full text of the articles retrieved following the title/abstract review.

We identified 413 relevant citations, of which 45 fulfilled the inclusion criteria (Figure 2; Table S2). Of the 45 data sources that were identified by the initial searches or recommended by the Literature Review Group, 17 articles were published in peer-reviewed journals; the remaining sources were published as InVS publications (n = 16), Institut Pasteur reports (n = 8), conference presentations (n = 3) or French Ministry of Health reports (n = 1). The study designs varied, but the majority (n = 22) were surveillance reports; the remaining data sources were outbreak reports (n = 2), prospective studies (n = 9), retrospective studies (n = 6), and ‘other’ (n = 6) (Table S2). A narrative synthesis of our findings is presented.

From the 45 publications, data were retrieved for the five territories as follows: 14 publications for French Guiana; 11 publications for Martinique; seven publications for Guadeloupe; six publications for Saint Martin; five publications for Saint Barthélemy; and 13 publications for French West Indies and French Guiana.

The data characteristics extracted for each study are presented in Table S2. Comprehensive data were lacking across the five territories for the entire review period and few data were available from non-epidemic years. Annual data collected by Cire AG were rarely made available to the public and therefore surveillance data collected during this review period were incomplete across the FTA. Available surveillance data from the five territories are shown in Table 1.

**Table 1:** Annual dengue epidemiology data from the French Territories of the Americas reported by the Cellule Regionale d' Épidémiologie Antilles Guyane (2000–2012).

Year	Suspected cases (n)	Incidence per 100,000 population	Probable cases (n)	Laboratory-confirmed cases (n)	Deaths	Case fatality rate %	Reference
**French Guiana**							
2002	2614	1517*	172	165			[35]
2003			44*	5			[60]
2004	3095	1678*	185	309			[32]
2005	4329	2269*	361	509			[32]
2006	6983	3391*	876	688	4	0.06*	[33]
2007	5475	2571*	194	205			[28]
2008	5456	2489*	134	928			[34]
2009	3387	1509*	189	1364	2	0.06*	[39]
2006–2010	37812	17317*		10724			[26]
2010	3446*	1505*		953*			[15]
**Martinique**							
2005	228	58*					[32]
2006	66	17*					[33]
2007	246	62*					[28]
**Guadeloupe**							
2005	516	1114*					[32]
2006	277	70*					[33]
2007	11549	2884*					[36]
2007	546	137*	30	315			[28]
2008	173	44*	5	22			[34]
2009	283	71*	4	69			[39]
2010	597	149*	3	399			[15]
**Saint Martin**							
2005	39	111*					[32]
2006	44	125*	14				[33]
2009				72			[39]
2010				174			[15]
**Saint Barthélemy**							
2009				194			[39]
2010				152			[15]

*Calculated from the data available in the publication.

### Epidemiology of dengue in French Guiana

#### Epidemics

The periodicity of dengue epidemics in French Guiana was less than 3 years, with five epidemics observed during the review period, occurring in 2001, 2004–2005, 2005–2006, 2009 and 2009–2010 (Table 2). The epidemics varied in duration from 18 weeks in 2004–2005 to 29–40 weeks in 2005–2006, and the start of the epidemics coincided with the rainy seasons in French Guiana. The largest epidemic was in 2005–2006, during which there were 13,700–16,200 suspected cases [10], [13], [27]–[29], with an incidence rate of 7300–9000 cases per 100,000 population [30].

**Table 2:** Dengue epidemiology during epidemic years in the French Territories of the Americas (2000–2012).

Timing of epidemic	Duration (weeks)	Incidence (suspected cases [n] per 100,000 population)	Laboratory-confirmed cases (n)	Sex ratio (males∶females)	Hospitalizations		Case fatality rate (%)	Severity rate (%)	Reference	Reference type
					n	Rate (%)				
**French Guiana**										
Apr–Aug 2001	21.7	>1800 *							[35]	Surveillance
Jun–Dec 2004, Jan–Jul 2005	18				86				[10]	Outbreak report
Nov 2005–Jun/Sep 2006	29–40	7300–9000*	204–2300		204–211	1.26–1.54**	0.02–0.03**	1^a^	[10], [13], [26]–[30], [33], [36], [38]	Outbreak report; surveillance; prospective study
Jan–Aug/Sep 2009	32–37	6193–6505**	4129		241–247	1.70	0.01**	0.90^a^	[13], [26], [39]	Surveillance
Dec 2009/Jan 2010–Sep/Oct 2010	41.6	4026–4105**	2431		89–114	1.20	0.01**	0.50^a^	[13], [15], [26]	Surveillance
**Martinique**										
Sep 2001–Jan 2002	17.4–26	6190–7400*		0.58**	3–424*	0.12–0.18*	0.013*–0.016**	0.3–1* c	[9], [13], [17], [38], [43], [46], [47]	Genetic study; surveillance; prospective study
Jun 2005–Mar/Apr 2006	22–47.6**	3700	205		194–>200		0.013*–0.028**	0.3* a	[13], [17], [30], [37], [38], [42], [43], [45]	Surveillance; prospective clinical study
Aug/Sep 2007–Jan 2008	20–22	4524–4526**	4445**		352	1.9–2.0	0.013*–0.022**	1.2* a	[13], [36], [42], [43], [48]	Surveillance; prospective observational study
Feb–Oct 2010	35.9	10,000	9659	1.12	635–672**	1.6*	0.042**–0.045*	0.2* b	[13], [42]	Surveillance
**Guadeloupe**										
Jul 2005–Jan/Jun 2006	21–52	2000*			82	0.70*	0.009**	0.40* a	[13], [17], [30], [36]–[38], [44]	Surveillance
Aug/Sep 2007–Dec 2007/Jan 2009	17–19	4744**			272	1.40*	0.02**	0.80–1.0* a	[13], [17], [36], [38], [44]	Surveillance
Nov 2009–Oct 2010	47	10,933**	6357		411–418	0.90	0.014**	0.30–0.36* a	[13],[17],[42]	Surveillance
**Saint Martin**										
Dec 2002–Jan 2003	8.7	88**	2						[49]	Outbreak report
Oct 2003–Aug 2004	30.3–47.9	511*–639**	108–136**		12–17	6.8**–7.6**	0.44**	0^d^	[49], [50]	Outbreak report; virus characterization study
Nov 2007–Apr 2008	23.7	5869**	335		22	1.03**			[51]	Surveillance
2008–2009 (No specific date available)		5444**						0.5^d^	[16]	Surveillance
Nov 2009–May 2010	21.9	5000*			20	1.11**		0.75^d^	[16]	Surveillance
**Saint Barthélemy**										
Dec 2002–Jan 2003	8.7	364**	6		6	0.67			[49]	Outbreak report
Jun 2006–Nov 2006	24.9	7269 **	147		147	16.5*			[37]	Surveillance
No date available		14,367**	218		218	24.5*			[51]	Surveillance
Nov 2007–Apr 2008	23.8	5841**	123		6	1.2**		1.2** d	[16], [51]	Surveillance
Nov 2009–Mar 2010	20.9	6000*			5	1.0**		0.6** d	[16]	Surveillance

WHO, World Health Organization.

*Estimated value in the publication.

**Calculated from the data available in the publication.

a According to WHO 1997 dengue case severity classification.

b According to WHO 2009 dengue case severity classification.

c According to WHO 1986 dengue case severity classification.

d Dengue case severity classification not identify.

Further data regarding dengue severity (hospitalizations, deaths, case fatality rate, severity rate and severity classifications), including data from non-epidemic periods, are provided in the accompanying supplementary information (Table S4). Further data regarding the sex distribution of dengue, including data from non-epidemic periods are provided in the accompanying supplementary information (Table S5).

#### Dengue virus serotype distribution

**Figure 3:**
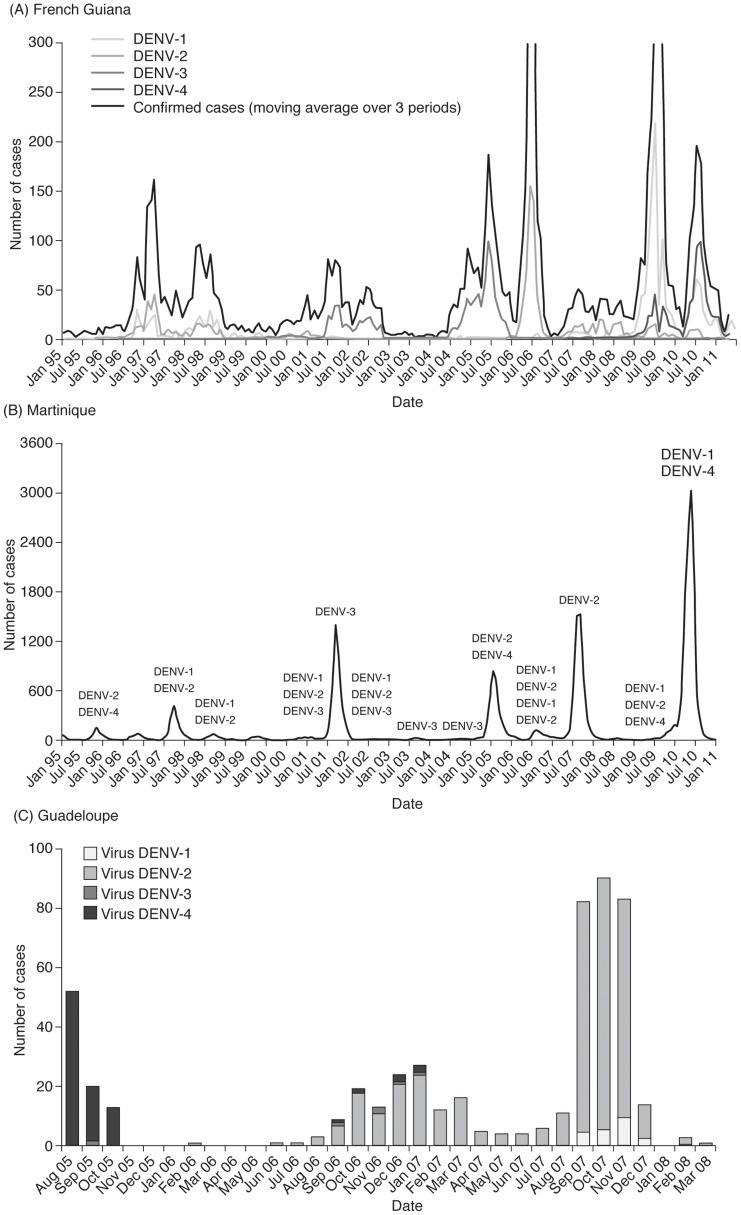
Distribution of dengue virus serotypes in (A) French Guiana, (B) Martinique and (C) Guadeloupe, 2000–2011. All four DENV serotypes have been in circulation in French Guiana and Martinique throughout the review period and from 2005 in Guadeloupe. The predominant serotypes varied over time, and shifts in predominant serotypes were concomitant with the reported epidemics. In all three territories, more than one DENV serotype was usually in circulation at any one time. In French Guiana co-circulation of all four serotypes was observed during the 2004–2005 epidemic, as well as in 2005, 2006 and 2008 and in Martinique, co-circulation of all four serotypes was observed in 2005–2006. (A) French Guiana. Figure reproduced from Quénel P, Rosine J, Cassadou S, Ardillon V, Blateau A, et al. (2011) Epidémiologie de la dengue dans les départements français d'Amérique. Bull Epidemiol Hebd 33–34: 358–363 [13], with permission from Bulletin Epidémiologique Hebdomadaire, Institut de Veille Sanitaire. (B) Martinique. Figure reproduced from Quénel P, Rosine J, Cassadou S, Ardillon V, Blateau A, et al. (2011) Epidémiologie de la dengue dans les départements français d'Amérique. Bull Epidemiol Hebd 33–34: 358–363 [13], with permission from Bulletin Epidémiologique Hebdomadaire, Institut de Veille Sanitaire. (C) Guadeloupe. Figure reproduced from Quénel P, Rosine J, Ardillon V, Cardoso T, Cassadou S, et al. (2008) Vers une hyperendémicité de la dengue dans les Antilles françaises. 9es Journées Nationales d'Infectiologie, 4–6 June 2008, Marseille, France [59], with permission from Philippe Quénel.

All four DENV serotypes have been in circulation in French Guiana throughout the review period (Table S3). The predominant serotypes varied over time, and shifts in predominant serotypes were concomitant with the reported epidemics (Figure 3A). With the exception of 2003 when circulation of DENV-1 was predominant (60%), DENV-1 was present at low levels until 2006 (range: 0.9–4.4%), after which its prevalence increased (range: 22.0–71.7%). During the review period, DENV-2 was not isolated until 2005, it was associated with the 2005–2006 epidemic and was predominant until 2008, after which it fell to low levels (range: 5.4–6.1%). DENV-3 circulated at high levels until 2005 (range: 40.0–98.6%) and was associated with the 2001 and 2004–2005 epidemics [10], after which it fell to low levels (0–4.9%). DENV-4 circulated at low levels from 2004 until 2008 (range: 0–12.3%), increased in 2009 (22.2–22.7%) and became predominant in 2010 (57.2%). Phylogenetic analyses of two DENV-4 strains isolated in French Guiana in 2004 and 2005 showed that they belonged to DENV-4 genotype II [31].

More than one DENV serotype was usually in circulation. Co-circulation of all four serotypes was observed during the 2004–2005 epidemic [13], [32], as well as in 2005, 2006 and 2008 [32]–[34]. From 2006, there were two main serotypes in circulation: the predominant serotype and a second main co-circulating serotype (Table S3).

#### Dengue severity

In French Guiana, data regarding the severity of dengue, including hospitalizations, deaths, case fatality rates, reports of severe cases of dengue and severity rates (ratio of the number of severe forms to the estimated number of dengue-like syndromes) were reported throughout the review period, with varying levels of information available for each epidemic (Table S4). The severity of dengue varied over time, with the most severe epidemic reported in 2005–2006.

In 2001 there were no reports of DHF or DSS [35]. The 2004–2005 epidemic was reported as being of moderate magnitude [13], with 86 hospitalizations [10]. The 2005–2006 epidemic was characterized by its magnitude and the high number of hospitalized cases observed [13]. During this epidemic, there were 211 hospitalizations [30], and the hospitalization rate was estimated at 1.3–1.5%. This epidemic was associated with a high number of severe dengue cases (27 DHF and 100–137 DSS and ‘other’) with a severity rate of 1.0% [10], [27], [30], [33]. There were four deaths and an associated case fatality rate of 0.02–0.06% [27]–[30], [36]–[38]. During the 2009 epidemic, there were 241–247 hospitalizations [26], [39] and the hospitalization rate was estimated at 1.70%. There were 129 severe cases and a severity rate of 0.90% [13], [26], [39], with two dengue-related deaths and an estimated case fatality rate of 0.01–0.06% [13], [26], [39]. The 2009–2010 epidemic was associated with the lowest reported severity rate (0.5%), with 36 severe cases, three of which were DSS [13], [15], [26]. There were 89–114 hospitalizations and a hospitalization rate of 1.20% [13], [15], [26]. One dengue-related death was reported and the case fatality rate was estimated at 0.01% [13], [15], [26].

#### Age distribution of dengue

Dengue appeared to affect adolescents and adults rather than young children. During the 2005–2006 epidemic, 62.0% of suspected cases and hospitalizations were in those aged >15 years, but three of the four reported deaths occurred in those aged <15 years [10], [29]. In a regional study conducted in the municipality of Maripasoula during the 2005–2006 epidemic, the highest prevalence of dengue was seen in those aged ≥55 years or 11–15 years [40]. Adolescents and adults were also shown to be more affected by dengue in 2008–2010, with 65.0% of hospitalizations in those aged >15 years [41].

#### Sex distribution of dengue

Of the five territories, data for the sex distribution of dengue were most abundant from French Guiana and were available for 2002 and 2005–2010 (Table S5). From the available data, slightly more men than women appeared to be affected by dengue (sex ratio males∶females ranged from 0.99–1.22). By contrast, a study conducted in Maripasoula in 2005–2006 and a hospital-based study from 2008–2010 suggested that women were more affected than men (sex ratio of males∶ females 0.90 and 0.72, respectively [40], [41].

#### Seroprevalence

Seroprevalence data in French Guiana were available only from one study conducted across six maternity units during the 2005–2006 epidemic. Of the 586 women included in the study, 92.0% were seropositive for a flavivirus. However, these results are difficult to interpret due to the mandatory vaccination against yellow fever and circulation of other flaviviruses in the region [27].

#### Seasonality, climate and environment

Dengue in French Guiana was without marked seasonal variation. The Cayenne peninsula municipalities (Cayenne, Remire-Montjoly and Matoury) were often the origin for the emergence and re-emergence of DENV serotypes [10]. The first cases reported during epidemics were usually from Cayenne, which is the main area for contact with visitors to the country due to the presence of the only international airport in the region [10].

### Epidemiology of dengue in Martinique and Guadeloupe

#### Epidemics

The timing and duration of the epidemics during the review period in Guadeloupe (2005–2006, 2007–2008 and 2010) were similar to those in Martinique (2001–2002, 2005–2006, 2007–2008 and 2010) (Table 2). The magnitude of the 2005–2006 epidemics was similar between Martinique and Guadeloupe, with an incidence rate of 3700 and 2000 suspected cases per 100,000 population, respectively [13], [30], [42]. The magnitude of the 2007–2008 epidemic was similar in Guadeloupe and in Martinique, with 4744 and 4524–4526 suspected cases per 100,000 population, respectively [13], [17], [36], [38], [42]–[44]. The largest of the epidemics in both territories were in 2010, first starting in Guadeloupe in November 2009, lasting 47 weeks until October 2010, followed by Martinique in February 2010, lasting 36 weeks until October 2010 [17], [42]. The magnitude of the 2010 epidemics was similar between these two close territories, with 40,000 and 44,000 suspected cases and an incidence rate of 10,000 and 10,933 suspected cases per 100,000 population in Martinique and Guadeloupe, respectively [13], [17], [42].

#### Dengue virus serotype distribution

All four DENV serotypes were in circulation during the review period in Martinique and from 2005 in Guadeloupe (no serotype data were available prior to this) (Table S3). From 2000 to 2005, DENV-3 was predominant in Martinique, with a low circulation of DENV-2 from 2001 to 2002. From 2005, the patterns of serotype distribution in Martinique and Guadeloupe were similar: DENV-4 was predominant in 2005, DENV-2 became predominant in 2006 and DENV-1 became predominant in 2009. In both territories, more than one DENV serotype was usually in circulation at any one time (Figures 3B and 3C), and in Martinique, co-circulation of all four serotypes was observed in 2005–2006 [37], [45].

Genetic characterization of five DENV-3 strains isolated in 2000 (n = 2) and 2001 (n = 3) in Martinique showed that all the isolates clustered together and were grouped as DENV-3 genotype III [9]. Phylogenetic analysis of two DENV-4 strains isolated in Martinique and one strain isolated in Guadeloupe in the fourth quarter of 2004 showed that they all belonged to DENV-4 genotype II [31].

#### Dengue severity

There were 424 hospitalizations during the 2001–2002 epidemic in Martinique, with an estimated hospitalization rate of 0.12–0.18% (Table S4) [46]. The severity rate was 0.30% and there were three reported cases of DHF/DSS and 77 other severe cases according to the WHO 1986 classification [9], [13], [47]. There were four dengue-related deaths and a case fatality rate of 0.013%–0.016% [13].

During the 2005–2006 epidemic there were 194–>200 hospitalizations in Martinique [13], [30], [37] and 82 hospitalizations in Guadeloupe [13], [30]. The severity rates for Martinique (0.30%) and Guadeloupe (0.40%) were similar [13], [30], [38], [42]–[44]. The two territories used different dengue classifications during this period. According to the WHO 1997 classification, there were 40–48 severe dengue cases reported in Martinique, including six cases of DHF. In Guadeloupe, 39 severe cases were reported according to the InVS 1998 classification, including 15 cases of DHF. There were four dengue-related deaths in Martinique versus one death in Guadeloupe, and the case fatality rate was slightly higher in Martinique (0.013–0.028%) than in Guadeloupe (0.009%) [13], [45].

During the 2007–2008 epidemic there were 352 hospitalizations and an associated hospitalization rate of 1.9–2.0% [13], [36], [42], [43], [45] in Martinique, compared with 272 hospitalizations and a hospitalization rate of 1.4% in Guadeloupe [13]. The severity rates were estimated at 1.2% and 0.8–1.0% in Martinique and Guadeloupe, respectively. The number of severe reported cases (according to the WHO 1997 classification) were higher in Martinique (219 cases) than in Guadeloupe (159 cases) [13], [17], [38], [42]–[44]. The number of deaths and estimated case fatality rates were similar between the two territories (Martinique: four deaths and a case fatality rate of 0.013%–0.022%; Guadeloupe: three deaths and a case fatality rate of 0.020%) [13], [36], [42], [43].

The 2010 epidemic was characterized by its magnitude and duration. There were 635–672 hospitalizations and a hospitalization rate of 1.60% in Martinique [13], [42]. There were also a large number of hospitalizations in Guadeloupe (411–418), with an estimated hospitalization rate of 0.90% [13], [17]. There were 75 severe dengue cases and a severity rate of 0.20% in Martinique (according to the WHO 2009 classification). In Guadeloupe, there were 156–160 severe cases and the severity rate was 0.30–0.36% (according to the WHO 1997 classification) [13], [17], [42]. The number of dengue-related deaths and the case fatality rates were higher in Martinique than in Guadeloupe (Martinique: 17–18 deaths and a case fatality rate of 0.042%–0.045%; Guadeloupe: 5–6 deaths and a case fatality rate of 0.014%) [13], [17], [42], [45], [47].

#### Age distribution of dengue

During 2007, the percentages of laboratory-confirmed cases in Martinique were 33.9% in children and adolescents (aged ≤19 years), 56.4% in adults aged 20–59 years and 9.7% in older adults (aged ≥60 years) [42]. Of the four deaths during the 2007–2008 epidemic, three were in adults aged ≥60 years and one was an 11-year-old child [36]. The age distribution pattern of laboratory-confirmed dengue cases was similar in 2010 to that observed in 2007, 38.4% of cases were in those aged ≤19 years, 50.7% in adults aged 20–59 years and 11.0% in adults aged ≥60 years [42]. During the 2010 epidemic, 37% of hospitalizations and 30% of deaths were in children aged ≤15 years.

Data on the age distribution of non-hospitalized laboratory-confirmed cases of dengue in Guadeloupe were available for 2007 and 2010 and the patterns were comparable with those observed in Martinique. In Guadeloupe, 36.7% of laboratory-confirmed cases were reported in children aged ≤19 years in 2007, rising to 48.8% during the 2010 epidemic [17]. There were three deaths during the 2007 epidemic, all in children aged <15 years [36]. In adults aged 20–59 years, the percentage of laboratory-confirmed cases fell from 53.9% in 2007 to 41.6% in 2010 [17]. Older adults aged ≥60 years were least affected by dengue, with <10% of laboratory-confirmed cases reported in this age group in both years [17].

#### Sex distribution of dengue

Data regarding the sex distribution of dengue from Martinique were limited and none were available from Guadeloupe (Table S5). During the 2001–2002 epidemic in Martinique, a study conducted in children aged 0–16 years in the emergency department in Lamentin showed a male∶female sex ratio of 1.15 [47]. During the 2005–2006 epidemic, a predominance of women (estimated male∶female sex ratio of 0.76) was observed among the 389 suspected dengue cases in adults (aged ≥15 years) in the emergency department in Fort de France [45].

When retrospectively classified using the WHO 2009 classification, confirmed cases of dengue in adults (aged ≥14 years) in the emergency department in Fort de France showed a male∶female sex ratio of 0.87 [48].

#### Seasonality, climate and environment

Dengue in Martinique and Guadeloupe is present all year round with a seasonal variability in incidence [13]. The seasonality in Guadeloupe and Martinique is very similar [13], and epidemics usually occurred in July to January. The 2010 epidemic in Martinique started in February and was earlier than expected, related to unusual climatic conditions, including lower than average rainfall and higher than average temperatures [42].

### Epidemiology of dengue in Saint Martin and Saint Barthélemy

#### Epidemics

Between 2000 and 2012, coinciding epidemics occurred in Saint Martin and Saint Barthélemy in 2002–2003, 2007–2008 and 2009–2010. These epidemics were of similar magnitude in each territory and, where data were available, the timing and duration were also comparable (Table 2). Additional epidemics occurred in Saint Martin during 2003–2004 and 2008–2009, and in Saint Barthélemy during 2006 and 2006–2007.

In Saint Martin, epidemics increased in magnitude over time. Suspected cases rose from 31 in the 2002–2003 epidemic to approximately 2000 in the three epidemics occurring from 2007 onwards, and corresponding incidence rates rose from 88 per 100,000 population to over 5000 per 100,000 population [16],[49]–[51]. The largest epidemic was in 2007–2008, with 2130 suspected cases and an incidence of 5869 suspected cases per 100,000 population [51]. During the same period, the magnitude of the epidemics in Saint Barthélemy fluctuated. The smallest epidemic occurred in 2002–2003 with 30 suspected cases and an incidence of 364 suspected cases per 100,000 population [49]. The largest epidemic occurred in 2006–2007, with 1200 suspected cases and an incidence of 14,367 suspected cases per 100,000 population [51]. The 2007–2008 and 2009–2010 epidemics were similar to one another in magnitude, with 500 suspected cases and an incidence of approximately 6000 suspected cases per 100,000 population [16], [37], [51].

#### Dengue virus serotype distribution

For the periods with available data, a similar pattern of serotype distribution over time was observed in Saint Martin and Saint Barthélemy (See Matheus et al. [11] Figure 1 [http://www.ajtmh.org/content/86/1/159/F1.expansion.html] and Figure 2 [http://www.ajtmh.org/content/86/1/159/F2.expansion.html]).

Serotype data were available from Saint Martin for the period 2002 onwards (Table S3). During 2002–2005, DENV-3 was the only serotype in circulation [32], [49], [50]. In 2006, DENV-2 became predominant, with co-circulation of DENV-3 and DENV-4 [37]. From 2007 to 2008, DENV-1 was predominant, with co-circulation of DENV-2 during the 2008–2009 epidemic [11], [34], [39]. The predominant serotype switched back to DENV-2 in 2009, with co-circulation of DENV-4 during the 2008–2009 epidemic [39], and switched to DENV-1 in 2010, with co-circulation of DENV-2 [11], [15].

Little data on serotype distribution were available from Saint Barthélemy prior to 2007 (Table S3). DENV-3 was the only isolated serotype during the 2002–2003 epidemic [49]. From 2007 to 2010, DENV-1 was predominant with co-circulation of DENV-2. DENV-4 was present at low levels (0.3–1.3%) in 2008–2010.

Phylogenetic analysis of DENV-3 virus isolates from six patients in Saint Martin during the 2003–2004 outbreak showed that all six isolates were DENV-3 genotype III [50].

#### Dengue severity

Data regarding the severity of dengue in Saint Martin and Saint Barthélemy were limited. The severity rate in Saint Martin increased from 0% in the 2002–2003 epidemic to 0.75% in the 2009–2010 epidemic (Table 2) [16], [50]. By contrast, the hospitalization rate decreased between 2003–2004 and 2009–2010, from 6.8–7.6% to 1.11%, respectively [16], [49]–[51]. In comparison, the data available for Saint Barthélemy showed a decrease in severity, from a severity rate of 1.2% in 2007–2008, to 0.6% in 2009–2010 [16], [51]. Hospitalization rates varied between 2002–2003 and 2009–2010, ranging from 0.67% to 24.5% [16], [37], [49], [51].

During the 2007–2008 epidemic there were 22 hospitalizations in Saint Martin and six in Saint Barthélemy; corresponding hospitalization rates were 1.03% and 1.20% [16], [51]. Hospitalizations were similar during the 2009–2010 epidemic to the 2007–2008 epidemic, with 20 hospitalizations and a hospitalization rate of 1.11% in Saint Martin and five hospitalizations and a hospitalization rate of 1.0% in Saint Barthélemy [16]. There were 15 severe cases reported in Saint Martin compared with three severe cases in Saint Barthélemy (according to the WHO 1997 case definition) (Table S4).

#### Age distribution of dengue

Data on the age distribution of dengue were lacking. Data for Saint Martin were available for 2003–2004 from a community epidemiology study in three districts, which showed that suspected dengue cases were highest in the 20–39 year age group (42.9% of suspected cases) and lowest in those aged ≥60 years (7.1%) [49]. Data were also available for dengue hospitalizations in 2007–2008 and showed that the hospitalization rate was higher in adults (63.6%) than in children (36.4%) [51]. No data were available for the age distribution of dengue in Saint Barthélemy.

#### Sex distribution of dengue

Data regarding the sex distribution of dengue were available from one study in Saint Martin conducted in the districts of Baie Orientale, Cul de Sac and Mont O'Reilly during the 2003–2004 epidemic. The study showed that dengue was more prevalent in women than in men (sex ratio males∶females 0.70) [49] (Table S5). No data regarding sex distribution of dengue were available for Saint Barthélemy.

#### Seasonality, climate and environment

Dengue in Saint Martin and Saint Barthélemy is endemic, with transmissions reported all year round [50]. Epidemics were usually concomitant with heavy rainfall in May to November [50]. However, since 2001, epidemics were mainly reported between October and February [51].

## Discussion

This report provides a descriptive summary of the epidemiology of dengue in the FTA (Martinique, Guadeloupe, Saint Martin, Saint Barthélemy and French Guiana) for 2000–2012. Despite substantial gaps in epidemiological knowledge these data are important for assessing the epidemiological evolution both within and across territories and years.

Dengue is endemic throughout the FTA, although it appears that dengue in this region is evolving towards hyperendemicity. Such a transition is similar to the epidemiological patterns observed in neighbouring countries such as Brazil, where dengue is also moving towards a hyperendemic state [52].

The most intense epidemic years across the FTA occurred between 2006 and 2010 and were associated with predominance of DENV-1 and DENV-2. The epidemic years with the highest dengue incidence were 2006 in French Guiana, which was associated with DENV-2; 2010 in Martinique-Guadeloupe, which was associated with DENV-1; and 2006–2007 for Saint Barthélemy and 2007–2008 for Saint Martin, which were both associated with DENV-1. The epidemic years with the highest severity rates were 2006 in French Guiana (1.0%), associated with DENV-2; 2007–2008 in Martinique (1.2%) and 2007 in Guadeloupe (0.8–1.0%), both associated with DENV-2; 2009–2010 in Saint Martin (0.75%), associated with DENV-2; and 2007–2008 in Saint Barthélemy (1.2%), associated with DENV-1.

Epidemics usually occurred after a shift in the predominant serotype was observed, when non-immune populations (e.g., tourists, people newly settled in the FTA, or people not previously exposed to the circulating serotypes) were exposed to the new serotype through human movements inside territories or across neighbouring countries. We found that epidemic characteristics such as the duration of epidemics and circulating serotypes were shared between the close geographic territories of Martinique and Guadeloupe, and Saint Martin and Saint Barthélemy.

### Gaps in epidemiological knowledge

There were substantial gaps in epidemiological knowledge regarding DENVs in the region. In particular, information regarding DENV serotypes was limited during inter-epidemic periods, with the exception of French Guiana where the National Reference Center of arboviruses of the Pasteur Institute is located. Additionally, the available data were not always representative of the population as a whole. During epidemics, most of the analysed samples were taken from hospitalized cases, so circulating serotypes in non-severe cases were not well documented. Moreover, data regarding DENV genotypes circulating in the FTA were not routinely available. In addition, the impact of population movements (e.g., due to tourism and immigration) on the circulating serotypes and local serotype evolution in these highly touristic areas was not available, but would be useful to understand and document the epidemiological situation.

The populations at highest risk of contracting dengue over time were not identified from the data available from this literature search and analysis for any of the five territories. Data regarding the age distribution of dengue were often lacking in surveillance reports and epidemiological studies. Furthermore, seroprevalence for dengue is not documented in the FTA and the only available data were from an epidemiological study in pregnant women across six maternity units in French Guiana [53]. This lack of data limits the potential to make comparisons and draw conclusions over time, across territories and between different age groups. Further epidemiological studies are required to examine these parameters, the results of which will inform public health officials of the populations at highest risk of contracting dengue and help to strengthen control measures.

Although indicators of severe dengue are well documented, the mechanisms of severity are unclear and are likely to be multifactorial. Possible risk factors for severe disease include racial/ethnic factors and comorbidities, including sickle-cell disease [47], [54], [55]. Further studies to understand the role of ethnicity on the dynamics and severity of dengue in the FTA would be informative in these populations of mixed ethnicity. Collection of seroprevalence data would be useful, as secondary DENV infections are a known risk factor for severe disease [56].

The majority of data were reported during epidemics; further reporting of data during inter-epidemic periods would help to document the evolution towards hyperendemicity in the FTA. Other interesting data not covered in this review are data relating to the interaction between the environment and the host vector, which would help us to better understand the factors and conditions that contribute to the spread of the disease.

Surveillance systems across the FTA have made continuous improvements over the last decade and further improvements will help to fill the gaps in epidemiological knowledge. In 2004, the CNR and Cire AG set up the transport of biological samples between the French West Indies and French Guiana to facilitate virological surveillance [28]. Since 2008, all laboratories in French Guiana have adopted the early diagnosis test for DENV based on detection of the NS1 antigen and anti-DENV IgM. Furthermore, since 2008, collection of blood on filter paper [11], [18] has overcome the constraints of transport and preservation of biological samples from Saint Martin and Saint Barthélemy, as well as remote health centres in French Guiana, to assure follow-up of patients [33] and identification of the circulating serotypes in a more systematic way. In the French West Indies, new technologies such as the use of data modelling using frequency analysis (Serfling periodic regression) or temporal analysis (Box and Jenkins method) to determine thresholds for dengue-like syndrome cases and laboratory-confirmed cases have allowed detection of unusual dengue activity against endemic background noise [26], [57], [58]. The sensitivity, specificity and predictive positive value of these thresholds have aided the detection of pre-epidemic and epidemic alerts, allowing for better preparation for the implementation of control measures. In summary there is a need for epidemiologic and seroprevalence data (both during epidemic and inter-epidemic periods and grouped by age and sex) that is representative of the populations as a whole. Other specific gaps identified include: DENV serotype distribution data (in non-severe dengue [i.e. non-hospitalized cases] and impact of population movements); DENV genotype characterization; and interaction between the environment and the host vector.

### Strengths and limitations

There were several limitations of this study. Few epidemiological studies were published between 2000 and 2012 and the studies included in this review may be subject to publication bias, as the protocol mainly captured published studies. There was heavy reliance on the accuracy of the data reported to the surveillance systems and it was likely that some variability existed in the reporting of data across the territories. Furthermore, different case definitions for dengue were used across the territories over time, and changes to the surveillance system, including adoption of the laboratory confirmation test and changes in sampling methods and shipment of samples for testing, made comparisons across studies difficult. Despite these limitations, there were also several important strengths of this study, which lie in the methodology and synthesis of the results. Our literature review was thorough. We screened over 400 articles to identify relevant publications, and the active surveillance system produces consistent data across the five territories, which are made publically available.

### Conclusions

Dengue is a major public health concern in French Guiana, Martinique, Guadeloupe, Saint Martin and Saint Barthélemy. The epidemiology of dengue in this region has changed between 2000 and 2012 and is characterized by a marked increase in the frequency, magnitude and severity of epidemics across all the territories, an increase in the co-circulation of serotypes and the evolution to a hyperendemic state. Countries sharing geographical and environmental characteristics (e.g., Martinique and Guadeloupe, and Saint Barthélemy and Saint Martin) also shared characteristics in dengue epidemiology and its evolution over time, including timing of epidemics and circulating serotypes. Epidemiological and virological surveillance of dengue in the FTA is evolving, and further improvements to the knowledge of the disease in these territories will improve anticipation of epidemics and aid implementation of control measures.

## Supporting Information

Table S1 **Databases, on-line sources and grey literature searched for publications relating to the epidemiology of dengue in the French Territories of the Americas.**
(PDF)

Table S2 **Data characteristics extracted for each study included in the review.**
(PDF)

Table S3 **Dengue virus serotype distribution in the French Territories of the Americas (2000–2012).**
(PDF)

Table S4 **Dengue severity data in the French Territories of the Americas (2000–2012).**
(PDF)

Table S5 **Sex distribution of dengue in the French Territories of the Americas (2000–2012).**
(PDF)

Checklist S1 **PRISMA checklist.**
(PDF)
