# Supplementary material for: Recent Epidemiological Trends of Dengue in the French Territories of the Americas (2000–2012): A Systematic Literature Review
Source: PLoS Negl Trop Dis. 2014 Nov 6;8(11):e3235. doi: 10.1371/journal.pntd.0003235 (PMC4222734; doi:10.1371/journal.pntd.0003235)
Supplement: Table S1 — Databases, on-line sources and grey literature searched for publications relating to the epidemiology of dengue in the French Territories of the Americas. (PDF) [file pntd.0003235.s001.pdf]

Table S1. Databases, on-line sources and grey literature searched for publications relating to the epidemiology of dengue in the French Territories of the Americas.

**Source**	**Website**
United States National Library of Medicine and the National Institutes of Health Medical Database (PubMed)	http://www.ncbi.nlm.nih.gov/pubmed/
MedLine	
Excerpta Medica Database (EMBASE)	
World Health Organization Library database (WHOLIS)	http://www.who.int/library/databases/en/
WHO Regional Database: the Virtual Health Library (VHL)	http://www.who.int/library/databases/paho/en/
Scientific Electronic Library Online (SciELO)	http://www.scielo.org/php/index.php?lang=en
Latin American and Caribbean Health Science Literature (LILACS)	http://lilacs.bvsalud.org/en/
Institut de Veille Sanitaire (InVS) library database	http://opac.invs.sante.fr
Institut de recherche pour le développement (IRD) database	http://horizon.documentation.ird.fr/exl-php/cadcgp.php
Institut Pasteur - French Guiana	http://www.pasteur-cayenne.fr/spip/spip.php?article31
Institut Pasteur - Guadeloupe	http://www.pasteur-guadeloupe.fr/
Centres Nationaux de Référence (CNR) des Arbovirus	http://www.pasteur.fr/ip/easysite/pasteur/fr/sante/centres-nationaux-de-reference-et-centres-collaborateurs-de-l-oms
Centres Nationaux de Référence (CNR) des Fièvres Hémorragiques Virales	http://www.pasteur.fr/ip/easysite/pasteur/fr/sante/centres-nationaux-de-reference-et-centres-collaborateurs-de-l-oms
Ministère des Affaires sociales et de la Santé	http://www.sante.gouv.fr/
Direction de la Santé et du Développement Social (DSDS)	http://www.martinique.sante.gouv.fr/; http://www.guadeloupe.sante.gouv.fr/;

Martinique, Guadeloupe and French Guiana*	http://www.guyane.sante.gouv.fr/
Agence Régionale de Santé (ARS) Martinique, Guadeloupe and French Guiana	http://www.ars.guyane.sante.fr/Internet.guyane.0.html; http://www.ars.guadeloupe.sante.fr/Internet.guadeloupe.0.html; http://www.ars.martinique.sante.fr/Internet.martinique.0.html
Observatoires Régionaux de la Santé	http://www.orsag.fr/; http://www.ors-guyane.org/; http://www.ors-martinique.org/
Centres Hospitaliers (Centre Hospitalier Universitaire de Fort-de-France ; Centre Hospitalier Universitaire de Pointe-à-Pitre Abymes ; Centre Hospitalier de Cayenne Andrée Rosemon)	http://www.chu-fortdefrance.fr/; http://www.chu-guadeloupe.fr/; http://www.ch-cayenne.net/
Bulletin d'alerte et de surveillance Antilles Guyane (BASAG)	http://www.invs.sante.fr/publications/basag/index.html
Bulletin de Veille Sanitaire Antilles Guyane (BVS)	http://www.invs.sante.fr/publications/bvs/antilles_guyane/index.html
Bulletin Epidémiologique Hebdomadaire (BEH)	http://www.invs.sante.fr/beh/
Catalogue Sudoc: la bibliographie nationale des thèses en France	http://www.sudoc.abes.fr/
Theses.fr	http://www.theses.fr/

* Direction de la Santé et du Développement Social (DSDS) Martinique, Guadeloupe and French Guiana database and web links were available at the time that the literature search was performed, but are no longer available.
