# Supplementary material for: Recent Epidemiological Trends of Dengue in the French Territories of the Americas (2000–2012): A Systematic Literature Review
Source: PLoS Negl Trop Dis. 2014 Nov 6;8(11):e3235. doi: 10.1371/journal.pntd.0003235 (PMC4222734; doi:10.1371/journal.pntd.0003235)
Supplement: Table S2 — Data characteristics extracted for each study included in the review. (PDF) [file pntd.0003235.s002.pdf]

Table S2. Data characteristics extracted for each study included in the review.

Citation	Region or geographical area	Study design	Data period: date range: year	Study details (if applicable)		Summary of data presentation or results/conclusion
				No. patients/population studied (M:F)	Age range	
Peyrefitte et al., 2003 [9]	Martinique	Genetic characterization of newly re-introduced DENV-3 in Martinique	2000–2001	5 isolates		97 suspected cases, all of which were laboratory-confirmed; 97 cases of dengue fever (WHO 1986 classification)
Mattera et al., 2006 [10]	French Guiana	Outbreak report	Up to 30 June 2006			French Ministry of Health report detailing the epidemiology of dengue in French Guiana up to 2006, providing information on timing of dengue epidemics, the contribution of environmental and climatic factors, serotype distribution, suspected cases, incidence rates, hospitalizations, hospitalization rate and dengue severity. Included data from hospital-based studies
Matheus et al., 2012 [11]	Saint Martin and Saint Barthélemy	Prospective syndromic and virological surveillance of dengue in Saint Martin and Saint Barthélemy using blood samples absorbed on to filter paper	October 2008 (week 39)–December 2010 (week 50)	666 samples (288 for Saint Martin and 378 for Saint Barthélemy)		Trends in number of laboratory-confirmed cases of dengue and DENV serotype distribution in Saint Martin and Saint Barthélemy between 2008–2010
Quenel et al., 2011 [13]	French West Indies, French Guiana	Surveillance study	2001–2010			Surveillance report detailing the epidemiology of dengue in the French West Indies between 1991–2010, highlighting the dramatic increase in incidence of dengue over time
National Reference Center, 2011 [15]	FTA	Surveillance report	2010			Surveillance report detailing epidemiology of dengue, including suspected cases, probable cases, laboratory-confirmed cases, incidence rates and DENV serotype distribution

Citation	Region or geographical area	Study design	Data period: date range: year	Study details (if applicable)		Summary of data presentation or results/conclusion
				No. patients/population studied (M:F)	Age range	
Larrieu et al., 2010 [16]	Saint-Martin, Saint Barthélemy	Surveillance study	2009–2010			Report characterizing the epidemiology of dengue during 2009–2010, including timing and duration of epidemics, suspected cases, laboratory-confirmed cases, incidence rates, hospitalizations, hospitalization rates, severity and DENV serotype distribution
Chappert et al., 2011 [17]	Guadeloupe	Surveillance report	December 2009–October 2010			43,800 suspected cases; 6317 laboratory-confirmed cases; DENV serotype distribution: DENV-1, 98.5%; DENV-4, 1.5%
Cassadou et al., 2011 [18]	Saint Barthélemy	Surveillance report	2005–2010			Report detailing the evolution of dengue surveillance in Saint Barthélemy and providing data on the epidemiology of dengue (suspected cases, laboratory-confirmed cases and incidence rates)
Flamand et al., 2011 [26]	French Guiana	Surveillance methodology	2006–2010			Report on the validity and performance of the surveillance system implemented in 2006. Presentation of data collected between 2006–2010: 37,812 suspected cases; 10,724 laboratory-confirmed cases
Meynard et al., 2009 [27]	French Guiana	Prospective seroprevalence study in pregnant women	September–December 2006	689	13.9–43.9 years	92% of women had been exposed to a flavivirus in their natural environment; 1.9% of women were probable cases of dengue
National Reference Center, 2008 [28]	FTA	Surveillance report	2007			Surveillance report detailing epidemiology of dengue, including timing and duration of epidemics, suspected cases, probable cases, laboratory-confirmed cases, incidence rates and DENV serotype distribution
Delattre et al., 2007 [29]	Cayenne, French Guiana	Prospective, transversal, observational study in hospitalized children	November 2005–December 2006	125 cases	Paediatric population	125 suspected cases; 76 laboratory-confirmed cases; 1 death; 49 non-severe cases (WHO 1997 classification)
Rosine et al., 2007 [30]	Martinique, Guadeloupe, French Guiana	Surveillance study	2005–2007			Description of the 2005–2006 and 2006–2007 dengue outbreaks, including number of suspected cases, serotype distribution, number of deaths, number of hospitalizations, number of severe cases and age distribution among hospitalized cases

Citation	Region or geographical area	Study design	Data period: date range: year	Study details (if applicable)		Summary of data presentation or results/conclusion
				No. patients/population studied (M:F)	Age range	
Dussart et al., 2006 [31]	French West Indies, French Guiana	Phylogenetic analyses of DENV-4 strains	2004–2005	DENV-4 strains from human sera: 6 from French Guiana in 1993 and 1995; 2 from French Guiana in 2004 and 2005; 2 from Martinique in 2004; 1 from Guadeloupe in 2004		Phylogenetic analyses of two DENV-4 strains isolated in French Guiana in 2004 and 2005 showed that they belonged to DENV-4 genotype II. Phylogenetic analysis of two DENV-4 strains isolated in Martinique and one strain isolated in Guadeloupe in the fourth quarter of 2004 showed that they all belonged to DENV-4 genotype II.
Institut Pasteur de la Guyane, 2008 [32]	FTA	Surveillance report	2004–2005			Surveillance report detailing epidemiology of dengue, including suspected cases, probable cases, laboratory-confirmed cases, incidence rates and DENV serotype distribution
National Reference Center, 2007 [33]	FTA	Surveillance report	2006			Surveillance report detailing epidemiology of dengue, including suspected cases, probable cases, laboratory-confirmed cases, incidence rates, deaths, case fatality rates, DENV serotype distribution
National Reference Center, 2009 [34]	FTA	Surveillance report	2008			Surveillance report detailing epidemiology of dengue, including suspected cases, probable cases, laboratory-confirmed cases, incidence rates and DENV serotype distribution
Institut Pasteur de la Guyane, 2008 [35]	FTA	Surveillance report	2001			Surveillance report detailing epidemiology of dengue, including timing and duration of epidemics, suspected cases, probable cases, laboratory-confirmed cases, incidence rates, severity and DENV serotype distribution
Césaire et al., 2008 [36]	French West Indies, French Guiana, Saint Martin and Saint Barthélemy	Review article				Presentation of epidemiological characteristics of dengue epidemics across the FTA including epidemic timings and durations; numbers of cases, incidences, hospitalizations, deaths, lethality rates

Citation	Region or geographical area	Study design	Data period: date range: year	Study details (if applicable)		Summary of data presentation or results/conclusion
				No. patients/population studied (M:F)	Age range	
Institut de Veille Sanitaire, 2006 [37]	Martinique, Guadeloupe, Saint Martin, Saint Barthélemy	Surveillance study	2005–November 2006			Report characterizing the epidemiology of dengue during 2005–2006, including timing of epidemics, suspected cases, laboratory-confirmed cases, incidence rates, hospitalizations, hospitalization rates and DENV serotype distribution
Rosine et al., 2007 [38]	French Guiana, Martinique, Guadeloupe	Surveillance study	1995–2007			Epidemiology of dengue, including timing and duration of epidemics, suspected cases, laboratory-confirmed cases, deaths, severity and DENV serotype distribution
National Reference Center, 2010 [39]	FTA	Surveillance report	2009			Surveillance report detailing epidemiology of dengue, including timing and duration of epidemics, suspected cases, probable cases, laboratory-confirmed cases, incidence rates, deaths, case fatality rates, and DENV serotype distribution. Included data collected by using a new blood sampling method (capillary blood sampling on filter papers) in landlocked municipalities in French Guiana
Meynard et al., 2009 [40]	Maripasoula (high Maroni area), French Guiana	Retrospective outbreak investigation of dengue fever in Maripasoula	November 2005–February 2006	528 individuals (486 in their houses and 42 in their work places). People interviewed in their house, sex ratio M:F 0.93. People interviewed at their working place, sex ratio M:F 1.0	People interviewed in their house, average age: 17.2 years (range: 1–996 months; median: 156 months). People interviewed at their working place, average age: 33.7 years (range: 252–624 months; median: 426 months)	127 suspected cases; 4 probable dengue infection cases; 2 laboratory-confirmed cases; M:F sex ratio: 0.9; DENV serotype distribution: DENV-2, 100%

Citation	Region or geographical area	Study design	Data period: date range: year	Study details (if applicable)		Summary of data presentation or results/conclusion
				No. patients/population studied (M:F)	Age range	
Djossou et al., 2011 [41]	French Guiana	Prospective hospital study	November 2008–June 2010	392: 42% males,58% females	<15y = 35%<br>>60y = 5%	18,400 suspected cases; incidence rate: 8205 per 100,000; 6,800 laboratory-confirmed cases; 392 hospitalizations (<15 years: 35%; >60 years: 5%); hospitalization rate: 2.13%; 3 deaths; lethality rate: 0.02%; severity rate 0.05% (according to WHO 1997 classification) or 0.59% (according to WHO 2009 classification), or 0.99% (according to InVS 1998 classification)
Rosine et al., 2011 [42]	Martinique	Surveillance study	2010			The epidemic occurred between February and October 2010, lasting for 36 weeks; 9,659 laboratory-confirmed cases; M:F sex ratio: 1.12; 635 hospitalizations (<1 year: 5.1%; 1–5 years: 9.1%; 6–10 years: 12.4%; 11–15 years: 10.4%; ≥16 years: 63.0%); 17 deaths (<15 years: 30.0%; ≥16 years: 70.0%)
Institut de Veille Sanitaire, 2008 [43]	Martinique	Surveillance study	2001–2008<br>(Focus 2007–2008)			Presentation of the 2007–2008 outbreak in Martinique, including the number of suspected cases, percentage of severe cases and number of deaths, plus the presentation of data on the epidemiology of dengue between 2001–2008, including suspected cases and laboratory-confirmed cases
Institut de Veille Sanitaire, 2008 [44]	Guadeloupe continentales et îles proches (Marie-Galante, la Désirade, les Saintes)	Surveillance study	August (Week 35) 2007–December (Week 52) 2007			19,000 suspected cases; 272 hospitalizations; severity rate: 0.8%; DENV-2 predominant serotype

Citation	Region or geographical area	Study design	Data period: date range: year	Study details (if applicable)		Summary of data presentation or results/conclusion
				No. patients/population studied (M:F)	Age range	
Thomas et al., 2008 [45]	Fort de France, Martinique	Prospective clinical study in the adult emergency department of a tertiary care University Hospital of Fort de France	June 2005–April 2006	146 confirmed dengue virus infections. Sex ratio M:F 0.76	Median: 35 years	389 suspected cases with M:F sex ratio of 0.76; 36 probable dengue cases; 110 laboratory-confirmed cases; 71 hospitalizations; 4 deaths; WHO 1997 severity classification: 91 typical dengue fever cases, 9 DHF cases, 2 DSS cases; 21 cases presented at least one typical feature of DHF or DSS and 23 cases with unusual manifestations; DENV serotype distribution: DENV-1, 0.9%; DENV-2, 35.5%; DENV-3, 5.5%; DENV-4 58.2%
Merle et al., 2004 [46]	Martinique	Retrospective, transversal study	2001–2002	682 people: 47% males, 53% females	Mean: 38.2 years	122 suspected cases; incidence rate: 7,400 per 100,000; 3 hospitalizations; hospitalization rate: 0.18%; M:F sex ratio: 0.58
Monnin et al., 2005 [47]	Lamentin, Martinique	Retrospective, observational study in the Department of Paediatrics (CH Hospitalier du Lamentin - Martinique)	August 2001–February 2002	n=58 confirmed cases. Sex ratio M:F 1.15	0–4 years: 39.7%; 5–9 years: 17.2%; 10–16 years: 43.1%	48 probable cases of dengue; 10 laboratory-confirmed cases; M:F sex ratio: 1.15; 47 hospitalizations; 0 deaths; 3 cases of DHF (WHO 1997 classification); DENV serotype distribution: DENV-3, 100%
Thomas et al., 2012 [48]	Fort de France, Martinique	Prospective observational study in the adult emergency department of a tertiary-care university hospital of Fort de France	1 January 2005–31 December 2010	715 patients: 332 male and 383 female	14–91 (median 35)	715 laboratory-confirmed cases of dengue; M:F sex ratio: 0.87; 204 hospitalizations; 1 death; 383 dengue fever cases, 279 severe dengue cases, 53 cases of DHF/DSS; DENV serotype distribution: DENV-1, 22.5%; DENV-2, 51.1%; DENV-3, 2.5%; DENV-4, 23.9%
Malon et al., 2004 [49]	Saint Martin, Saint Barthélemy	Outbreak investigation report	2002–2003; Week 40 2003–Week 02 2004	Community epidemiology investigation: 70 cases. Sex ratio M:F 0.7	Community epidemiology investigation: mean: 35 years (95% CI: 30.8–38.8); range: 3–81 years <20 years: 18.6%; 20–39 years: 42.9%; 40–59 years: 31.4%; >60	Characterization of the 2002–2003 epidemics in Saint Martin and Saint Barthélemy and 2003–2004 epidemic in Saint Martin, including data from a community epidemiology investigation conducted in three districts in Saint Martin

Citation	Region or geographical area	Study design	Data period: date range: year	Study details (if applicable)		Summary of data presentation or results/conclusion
				No. patients/population studied (M:F)	Age range	
					years: 7.1%	
Peyrefitte et al., 2005 [50]	Saint Martin	Virus characterization during an outbreak	December 2003–January 2004	Sera from 26 patients with dengue-like syndromes		180 suspected cases; 108 laboratory-confirmed cases; 12 hospitalizations; hospitalization rate: 6.8%; 12 cases of non-severe dengue; DENV serotype distribution: DENV-3, 100%
Institut de Veille Sanitaire, 2008 [51]	Saint Martin, Saint Barthélemy	Surveillance study	2007–2008			Report characterizing the epidemiology of dengue during 2007–2008, including timing of epidemics, suspected cases, laboratory-confirmed cases, incidence rates, hospitalizations, hospitalization rates, severity and DENV serotype distribution
Basurko et al., 2009 [53]	Saint Laurent du Maroni Hospital, French Guiana	Retrospective study in pregnant women infected with the dengue virus during pregnancy	1 January 1992–10 September 2006	53 pregnant women	15–35 years; average: 26.4 years. <21 years: 38%	53 laboratory-confirmed cases; 1 death; DENV serotype distribution: DENV-1, 25%; DENV-2, 70%; DENV-3, 5%; DENV-4 0%
Quénel et al., 2008 [59]	French West Indies and French Guiana	Surveillance study	1991–2008			Presentation highlighting the shift in distribution of DENV serotypes in Guadeloupe, 2005–2008
Institut Pasteur de la Guyane, 2008 [60]	FTA	Surveillance report	2002–2003			Surveillance report detailing epidemiology of dengue, including suspected cases, probable cases, laboratory-confirmed cases and DENV serotype distribution
Djossou et al., 2009 [NC][A]	French Guiana	Prospective, observational hospital-based study	2006	211 cases		376 suspected cases; 211 laboratory-confirmed cases (according to WHO 1997 classification: 22.3% dengue fever; 3.3% DSS; 12.8% DHF; 61.6% other severe cases)
Dussart et al., 2012 [NC][B]	Cayenne, French Guiana	Multinational, prospective clinical study	July 2006–June 2007	28 patients in French Guiana		9 index cases and 28 household members recruited, among which 3 convalescents, 13 symptomatic, 3 unapparent and 9 non-infected dengue cases were identified
Gustave et al., 2008 [NC][C]	Guadeloupe communes de Abymes, Gosier, Saint François et Saint Barthélemy	Transversal, entomo-epidemiological study	September 2006–March 2007	158	Mean: 34 years	49–260 laboratory-confirmed cases (inter-epidemic period)

Citation	Region or geographical area	Study design	Data period: date range: year	Study details (if applicable)		Summary of data presentation or results/conclusion
				No. patients/population studied (M:F)	Age range	
Institut de Veille Sanitaire, 2005 [NC][D]	Martinique	Surveillance study	1995–2005			Surveillance report detailing epidemiology of dengue from 1995 to 2005 in Martinique, including epidemics with number of laboratory-confirmed cases and DENV serotype distribution and the geographical distribution of laboratory-confirmed cases in 2005.
Institut de Veille Sanitaire, 2006 [NC][E]	Cayenne, St Laurent du Maroni, Kourou, Matoury, French Guiana	Retrospective mortality database analysis	1 January 2005–30 April 2006			The number of observed deaths in four cities (n=666) was compared with the expected number of deaths (n=838). No excess of mortality during the dengue outbreak was observed
Rosine et al., 2007 [NC][F]	Martinique	Summary of entomo-epidemiological studies	Inter epidemic season 2004–2005	126 investigations		During the inter-epidemic season of 2004–2005, 126 epidemiological investigations were conducted in 38 cluster of transmission. These investigations were conducted on 198 suspected cases reported by the surveillance system and allowed the identification of 101 additional cases. The proportion of dengue cases was higher in people who had lived in Martinique <5 years (18% vs. 5%)
Tran et al., 2004 [NC][G]	Iracoubo, French Guiana	Prospective, spatiotemporal dengue transmission study	April–November 2001	161 patients with suspected dengue cases		161 suspected cases; 22 laboratory-confirmed cases; 10 probable cases; DEN-V serotype distribution: DENV-1, 9.5%; DENV-3, 90.5%
Chappert et al., 2009 [NC][H]	Guadeloupe quartier Fond Budan, Baie-Mahault	Retrospective, entomo-epidemiological study	26 February 2009–15 April 2009			Entomo-epidemiological investigation following the hospitalization of children.<br><br>14 suspected cases and 4 laboratory-confirmed cases

BASAG, Bulletin d'alerte et de surveillance Antilles Guyane; CI, confidence interval; DENV, dengue virus; DHF, dengue haemorrhagic fever; DSS dengue shock syndrome; EMBASE, Excerpta Medica Database; FTA, French Territories of the Americas; InVS, Institut de Veille Sanitaire publications; M:F, male:female; WHO, World Health Organization.

NC: Not cited; the following sources are not cited in the review:

A. Djossou F, Leon L, Demar-Pierre P, Gonon S, Delattre P, et al. (2009) Pertinence des critères de sévérité observés au cours de l'épidémie de dengue DEN-2 en Guyane française en 2006 [Poster].
B. Dussart P, Baril L, Petit L, Beniguel L, Quang LC, et al. (2012) Clinical and virological study of dengue cases and the members of their households: the multinational DENFRAME Project. PLoS Negl Trop Dis 6: e1482.
C. Gustave J, Faure K, Florentine G, Philetas M, Synesius A, et al. (2008) Importance de la dengue dans les populations provenant de zones indemnes du virus. Bulletin d'Alerte et de Surveillance Antilles Guyane 4: 3–5.
D. Institut de Veille Sanitaire (2006) Impact de l'épidémie de dengue sur la mortalité en Guyane. Numéro spécial. Situation épidémiologique de la dengue dans les 3 DFA. Bulletin d'Alerte et de Surveillance Antilles Guyane 4: 7.
E. Institut de Veille Sanitaire (2005) Bilan de la surveillance de la dengue aux Antilles (situation à risque pour le prochain hivernage). Bulletin d'Alerte et de Surveillance Antilles Guyane 7: 1–7.
F. Rosine J, Chaud P, Yebakima A, Malon A, Martinon M, Yp-Tcha MM, et al. Enquêtes entomo-épidémiologiques autour des cas de dengue en Martinique -
Bilan et perspectives pour la surveillance et le contrôle des épidémies de dengue. 2007.
G. Tran A, Deparis X, Dussart P, Morvan J, Rabarison P, et al. (2004) Dengue spatial and temporal patterns, French Guiana, 2001. Emerg Infect Dis 10: 615–621.
H. Chappert J (2009) Investigation d'un foyer de dengue au quartier Fond Budan, Baie-Mahault, avril 2009. Bulletin de veille sanitaire Antilles-Guyane 9: 7–8.
