# Supplementary material for: Recent Epidemiological Trends of Dengue in the French Territories of the Americas (2000–2012): A Systematic Literature Review
Source: PLoS Negl Trop Dis. 2014 Nov 6;8(11):e3235. doi: 10.1371/journal.pntd.0003235 (PMC4222734; doi:10.1371/journal.pntd.0003235)
Supplement: Table S3 — Dengue virus serotype distribution in the French Territories of the Americas (2000–2012). (PDF) [file pntd.0003235.s003.pdf]

**Table S3. Dengue virus serotype distribution in the French Territories of the Americas (2000–2012).**

| Year | Region/ population | DENV-1 (%/+) | DENV-2 (%/+) | DENV-3 (%/+) | DENV-4 (%/+) | Reference |
|---|---|---|---|---|---|---|
| **French Guiana** | | | | | | |
| 2001–2002 | Territory | ++ | + | **+++** | | [1,2] |
| 2001 | Iracoubo town | 9.5** | 0** | **90.5**** | 0** | [3,4] |
| 2002 | Territory† | 1.4** | 0** | **98.6**** | 0 | [2,4] |
| 2003 | Territory† | **60**** | 0** | 40** | 0 | [4] |
| 2004 | Territory† | 4.4** | 0** | **95.3**** | 0.4** | [5] |
| 2004–2005 | Territory | + | | | + | [6] |
| | | ++ | + | **+++** | + | [1] |
| | | | | **96.5** | | [7] |
| 2005 | Territory† | 0.9** | 6.1** | **92.0**** | 0.9** | [5] |
| | Maripasoula town | 0** | **100**** | 0** | 0** | [8] |
| 2005–2006 | Territory | | +++ | + | + | [7] |
| 2006 | Territory† | 1.4** | **97.7**** | 0.8** | 0.2** | [9] |
| | Territory | ++ | **+++** | + | | [1,6,9-11] |
| | | | **+++** | | | [12] |
| 2007 | Territory† | 22.0** | **73.2**** | 4.9** | 0** | [11] |
| 2008 | Territory† | **48.8**** | 38.2** | 0.7** | 12.3** | [13] |
| 2009 | Territory† | 71.2 | 6.1 | 0 | 22.7 | [14] |
| | Territory | **71.7**/+++** | 6.1**/+ | | 22.2**/++ | [1,12,14] |
| | Antecume pata | **100**** | 0** | 0** | 0** | [14] |
| | Camopi | **100**** | 0** | 0** | 0** | [14] |
| | Gran Santi | **100**** | 0** | 0** | 0** | [14] |
| 2010 | Territory† | 37.4 | 5.4 | 0 | **57.2** | [15] |
| | Territory | ++ | + | | **+++** | [1,12] |
| **Martinique** | | | | | | |
| 1999–2001 | Marine nursery | | | **+++** | | [16] |
| 2001 | Territory | | | ++ | | [17] |
| | | | + | **+++** | | [1,18] |
| 2001–2002 | Territory | 0** | 0.8** | **99.3**/+++** | 0** | [16,19] |
| | Lamentin (0–16 years) | 0** | 0** | **100**** | 0** | [19] |
| 2005 | Territory† | 0** | 5.6** | 0** | **94.4**** | [5] |
| 2005–2010 | Adult emergency (≥14 years old) | 22.5 | **51.1** | 2.5 | 23.9 | [18] |
| 2005–2006 | Territory | 0 | 30.0 | 0 | **70.0** | [1,6,9,10,17,20] |
| | Adult emergency (≥15 years) | 0.9** | 35.5** | 5.5** | **58.2**** | [21] |
| 2006 | Territory† | 0** | **60**** | 0** | 40** | [9] |
| | Territory | 2.6**/+ | 28.6**/++ | **48.1**/++** | 20.8**/+ | [17] |
| | | 0 | **94.0** | 0 | 6 | [6] |
| 2007 | Territory† | 4.0** | **96.0**** | 0** | 0** | [11] |
| 2007–2008 | Territory | | ++ | | | [1,20,22] |
| 2009 | Territory | + | + | | + | [14] |
| 2010 | Territory | **>70/+++** | | | | [15] |
| | | **67.0/++** | | | 33.0/+ | [1,20] |
| | | **67.4**** | 1.0** | 0 | 31.6 | [15] |

| | | | | | | |
|---|---|---|---|---|---|---|
| **Guadeloupe** | | | | | | |
| 2005 | Territory† | 0** | 0** | 4.5** | __95.4**__ | [5] |
| | Territory | | + | | ++ | [1] |
| 2005–2006 | Territory | | | | __+++__ | [6] |
| | | | __95.0__ | | | [9,17] |
| 2006 | Territory† | 0** | __90.3**__ | 0** | 9.7** | [9] |
| | Territory | 0 | __86.0__ | 8.0 | 5.0 | [17] |
| | | 0 | __93.0__ | 0 | 7.0 | [9] |
| 2006–2007 | Territory | 0 | __52.0__ | 41.0 | 7.0 | [6] |
| 2007 | Territory† | 8.3** | __91.7**__ | 0** | 0** | [11] |
| | Territory | + | __+++__ | | | [23] |
| | | | __+++__ | | | [1] |
| 2008 | Territory† | __72.7**__ | 22.7* | 4.5** | 0** | [13] |
| 2009 | Territory† | __98.6**__ | 0** | 1.4** | 0** | [14] |
| | Territory | + | | | | [14] |
| 2009–2010 | Territory | __98.5__ | 0 | 0 | 1.5 | [24] |
| 2010 | Territory† | __98.5__ | 0.3** | 0** | 1.3** | [15] |
| | Territory | ++ | | | ++ | [1] |
| | | __>70__ | ND | ND | ND | [15] |
| **Saint Martin** | | | | | | |
| 2002–2003 | Territory | 0** | 0** | ++ | 0** | [25] |
| 2003 | Territory | 0** | 0** | ++ | 0** | [25] |
| | | 0** | 0** | ++ | 0** | [25] |
| 2003–2004 | Territory | 0** | 0** | __100**__ | 0** | [26] |
| | | 0** | 0** | __100__ | 0** | [25] |
| | Four districts | 0** | 0** | __100**/++__ | 0** | [25] |
| 2004 | Territory | 0** | 0** | ++ | 0** | [25] |
| 2005 | Territory† | 0** | 0** | __100__ | 0** | [5] |
| 2006 | Territory† | 0** | __100__ | 0** | 0** | [9] |
| | Territory | 0** | __70.0**__ | 20.0** | 10** | [17] |
| 2007–2008 | Territory | __100__ | 0** | 0** | 0** | [27] |
| 2008 | Territory | __80.0**__ | 20.0** | 0** | 0** | [13] |
| 2008–2009 | Territory | ++ | ++ | 0** | 0** | [14] |
| | | __75.7__ | 24.3 | 0 | 0 | [28] |
| 2008–2010 | Territory | __71.8__ | 26.6 | 0 | 1.6 | [28] |
| 2009 | Territory† | 26 | __67.9__ | | 5.7 | [14] |
| | Territory | 0** | ++ | 0** | 0**/++ | [14] |
| | | 20.0 | __60.0__ | 20.0 | 0 | [28] |
| 2009–2010 | Territory | 25.0 | 75.0 | 0** | 0** | [29] |
| | | 28.3 | 71.7 | 0 | 0 | [28] |
| 2010 | Territory† | __82.0__ | 17.4 | 0 | 0.6 | [15] |
| | Territory | __75.0__ | 25.0 | 0 | 0 | [28] |
| | | __98.2__ | 0.9 | 0 | 0.9 | [28] |
| | | 0** | ++ | 0** | 0** | [15] |
| **Saint Barthélemy** | | | | | | |
| 2002–2003 | Territory | | | ++ | | [25] |
| 2007–2008 | Territory | __71.4**__ | 28.6** | 0 | 0 | [27] |
| 2008 | Territory | __57.1__ | 42.9 | 0 | 0 | [28] |
| | | __78.6**__ | 21.4** | 0** | 0** | [13] |
| 2008–2009 | Territory | __91.9/+++__ | 8.1 | 0 | 0 | [14,28] |

| | | | | | | |
|---|---|---|---|---|---|---|
| 2008–2010 | Territory | **95.4** | 4.3 | 0 | 0.3 | [28] |
| 2009 | Territory† | **97.1** | 2.9 | 0 | 0 | [14] |
| | Territory | **98.8** | 1.2 | 0 | 0 | [28] |
| 2009–2010 | Territory | **97.4** | 2.6 | 0 | 0 | [28,29] |
| 2010 | Territory† | **99.2** | 0 | 0 | 0.7 | [15] |
| | Territory | **98.7** | 0 | 0 | 1.3 | [28] |

Predominant serotype in **bold**

DENV, dengue virus; ND, Not determined.

*Estimated value in the publication

**Calculated from the data available in the publication

†Annual data from the National Reference Center in Arbovirus Surveillance

+, ++ and +++: semi-quantitative estimation of the serotype distribution from low to numerous

**References**


1. Quenel P, Rosine J, Cassadou S, Ardillon V, Blateau A, et al. (2011) Epidémiologie de la dengue dans les départements français d'Amérique. Bull Epidemiol Hebd 33-34: 358-363.
2. Institut Pasteur de la Guyane (2008) Rapport annuel 2001. Available: http://www.pasteur-cayenne.fr/spip/IMG/pdf/rapport_IPG_2001.pdf Accessed: 19 November 2013
3. Tran A, Deparis X, Dussart P, Morvan J, Rabarison P, et al. (2004) Dengue spatial and temporal patterns, French Guiana, 2001. Emerg Infect Dis 10: 615-621.
4. Institut Pasteur de la Guyane (2008) Rapport annuel 2002-2003. Available: http://www.pasteur-cayenne.fr/spip/IMG/pdf/rapport_IPG_2002-2003.pdf Accessed: 19 November 2013
5. Institut Pasteur de la Guyane (2008) Rapport annuel 2004-2005. Available: http://www.pasteur-cayenne.fr/spip/IMG/pdf/rapport_IPG_2004-2005.pdf Accessed: 19 November 2013
6. Rosine J, Ardillon V, Cardoso T, Cassadou S, Léon L, et al. (2007) Épidémiologie de la dengue aux Antilles et en Guyane: analyse comparative des dernières épidémies, 2005-2006 et 2006-2007 [Poster]. Journées de veille sanitaire 2007, 29-30 November 2007, Paris, France.
7. Mattera M, Vernerey M, Quatresous I (2006) L'épidémie de dengue survenue en Guyane en 2006. Available: http://opac.invs.sante.fr/index.php?lvl=author_see&id=7450 Accessed: 19 November 2013
8. Meynard JB, Ardillon V, Venturin C, Ravachol F, Basurko C, et al. (2009) First description of a dengue fever outbreak in the interior of French Guiana, February 2006. Eur J Public Health 19: 183-188.
9. National Reference Center of arboviruses and virus influenzae (2007) Rapport CNR arbovirus et virus influenza, région Antilles Guyane - Année 2006. Available: http://www.pasteur.fr/ip/resource/filecenter/document/01s-00004f-0r2/ra-cnr-arbo-ipg-2006.pdf Accessed: 19 November 2013
10. Césaire R, Cabie A, Djossou F, Lamaury I, Beaucaire G, et al. (2008) Aspects récents de la dengue dans les départements français d'Amérique. Virologie 12: 151-157.
11. National Reference Center of arboviruses and virus influenzae (2008) Rapport CNR arbovirus et virus influenza, région Antilles Guyane - Année 2007. Available: http://www.pasteur.fr/ip/resource/filecenter/document/01s-00004f-0ps/ra-cnr-arbo-ipg-2007.pdf Accessed: 19 November 2013
12. Flamand C, Quenel P, Ardillon V, Carvalho L, Bringay S, et al. (2011) The epidemiologic surveillance of dengue fever in French Guiana: when achievements trigger higher goals. Stud Health Technol Inform 169: 629-633.
13. National Reference Center of arboviruses and virus influenzae (2009) Rapport CNR arbovirus et virus influenza, région Antilles Guyane - Année 2008. Available: http://www.pasteur.fr/ip/resource/filecenter/document/01s-00004f-0pt/ra-cnr-arbo-ipg-2008.pdf Accessed: 19 November 2013
14. National Reference Center of arboviruses and virus influenzae (2010) Rapport CNR arbovirus et virus influenza, région Antilles Guyane - Année 2009. Available: http://www.pasteur.fr/ip/resource/filecenter/document/01s-00004f-0r3/ra-cnr-arbo-ipg-2009.pdf Accessed: 19 November 2013
15. National Reference Center of arboviruses and virus influenzae (2011) Rapport CNR arbovirus et virus influenza, région Antilles Guyane - Année 2010. Available: http://www.pasteur-

cayenne.fr/spip/IMG/pdf/Rapport_annuel_CNRA_IPG_2010_web_vf.pdf Accessed: 19 November 2013
16. Peyrefitte CN, Couissinier-Paris P, Mercier-Perennec V, Bessaud M, Martial J, et al. (2003) Genetic characterization of newly reintroduced dengue virus type 3 in Martinique (French West Indies). J Clin Microbiol 41: 5195-5198.
17. Institut de Veille Sanitaire (2006) Expérience tirée des épidémies de Martinique en 2005 et Guyane en 2006. BVS Antilles Guyane 7: 1-12.
18. Thomas L, Moravie V, Besnier F, Valentino R, Kaidomar S, et al. (2012) Clinical presentation of dengue among patients admitted to the adult emergency department of a tertiary care hospital in Martinique: implications for triage, management, and reporting. Ann Emerg Med 59: 42-50.
19. Monnin M, M'bou F (2005) An epidemic of dengue fever in a department of paediatrics: Report on 58 cases in Lamentin (Martinique). Arch Pediatr 12: 144-150.
20. Rosine J, AdélaÏde Y, Anglio J, Blateau A, Bousser V, et al. (2011) Bilan de l'épidémie de dengue en Martinique, 2010. BVS Antilles-Guyane 9-10 (Novembre-Décembre 2011): 2-6.
21. Thomas L, Verlaeten O, Cabie A, Kaidomar S, Moravie V, et al. (2008) Influence of the dengue serotype, previous dengue infection, and plasma viral load on clinical presentation and outcome during a dengue-2 and dengue-4 co-epidemic. Am J Trop Med Hyg 78: 990-998.
22. Institut de Veille Sanitaire (2008) Premier bilan de l'épidémie de dengue 2007-08 en Martinique. Bulletin d'Alerte et de Surveillance Antilles Guyane 4: 10.
23. Institut de Veille Sanitaire (2008) Premier bilan de l'épidémie de dengue 2007 en Guadeloupe. Bulletin d'Alerte et de Surveillance Antilles Guyane 4: 9.
24. Chappert J, Agnès M, Cassadou S, Ginhoux L, de Saint-Alary F, et al. (2011) Bilan de l'épidémie de dengue en Guadeloupe, 2010. BVS Antilles-Guyane No. 9-10 (Novembre-Décembre): 11-15.
25. Malon A, Chaud P, Gustave J (2004) Epidémie de Dengue à Saint-Martin (Guadeloupe). Rapport d'investigation. Available: http://www.invs.sante.fr/publications/2004/dengue_guadeloupe/dengue.pdf Accessed: 19 November 2013
26. Peyrefitte CN, Pastorino BA, Bessaud M, Gravier P, Tock F, et al. (2005) Dengue type 3 virus, Saint Martin, 2003-2004. Emerg Infect Dis 11: 757-761.
27. Institut de Veille Sanitaire (2008) Bilan épidémiologique de la dengue dans les îles de Saint Martin et de Saint Barthélemy, saison 2007 - 2008. Bulletin d'Alerte et de Surveillance Antilles Guyane 6: 12-13.
28. Matheus S, Chappert JL, Cassadou S, Berger F, Labeau B, et al. (2012) Virological surveillance of dengue in Saint Martin and Saint Barthelemy, French West Indies, using blood samples on filter paper. Am J Trop Med Hyg 86: 159-165.
29. Larrieu S, Hanson S, Brin F, Chappert J, Cassadou S, et al. (2010) Bilan de la surveillance et du contrôle des épidémies de dengue à Saint-Martin et Saint-Barthélemy: saison 2009-2010. BVS Antilles-Guyane 5: 6-8.
