# Supplementary material for: Recent Epidemiological Trends of Dengue in the French Territories of the Americas (2000–2012): A Systematic Literature Review
Source: PLoS Negl Trop Dis. 2014 Nov 6;8(11):e3235. doi: 10.1371/journal.pntd.0003235 (PMC4222734; doi:10.1371/journal.pntd.0003235)
Supplement: Table S4 — Dengue severity data in the French Territories of the Americas (2000–2012). (PDF) [file pntd.0003235.s004.pdf]

Table S4. Dengue severity data in the French Territories of the Americas (2000–2012).

Year	Population	Hospitalizations n	Hospitalizations Rate (%)	Deaths (n)	Case fatality rate (%)	Severity rate (%)	Non-severe cases (n)	Non-severe cases + warning signs (n)	Dengue fever (n)	Severe cases (n)	DHF (n)	DSS (n)	Others (n)	Definition used	Reference
**French Guiana**															
2001	Territory										0	0		WHO 1997	[1]
2002	Territory										0		109	WHO 1997	[1]
2004–2005	Territory	86													[2]
2005–2006	Territory	211	1.27–1.54**	4	0.02–0.06**	1[a]					27	0–7	100–130	WHO 1997	[2-6]
	Hospitalized children (Cayenne)	125		1								1		WHO 1997	[6]
	Hospitalized	171		4			28%				15%		50%	INVS 1998	[2]
2006	Territory	204	1.26–1.30**	4	0.02–0.03**	1[a]				0–163	0–27		0–100	WHO 1997	[7-11]
	Hospitalized (Cayenne)	376							47 (22.3%)		27 (12.8%)	7 (3.3%)	130 (61.6%)	WHO 1997	[12]
2008–2010	Territory	392	2.13**	3	0.02**	0.59[b]/ 0.05[a]/ 0.99[c]	42	242		108				WHO 2009	[13]
									383		9				
							210			182				INVS 1998	
2009	Territory	241–247	1.70	2	0.01–0.06**	0.90[a]				129				WHO 1997	[8,11], [14]
2009–2010	Territory	89–114	1.20	1	0.01**	0.50[a]				0–36		0–3		WHO 1997	[8,11,15],
**Martinique**															
2001–2002	Territory	3	0.18*												[16]
	Territory	424*	0.12*												[16]
	Territory	217		4	0.013*–0.016**	0.3[d]				77	3			WHO 1986	[8,17-20]
	Lamentin (0–16 years										3		13	WHO 1997	[19]

Year	Population	Hospitalizations		Deaths (n)	Case fatality rate (%)	Severity rate (%)	Non-severe cases (n)	Non-severe cases + warning signs (n)	Dengue fever (n)	Severe cases (n)	DHF (n)	DSS (n)	Others (n)	Definition used	Reference
		n	Rate (%)												
2002	Territory					≈1*									[10]
2005–2006	Territory	194–>200		4	0.013*–0.028**	0.3*[a]			139	40–42	6			WHO 1997	[3,8,10,17,18,21-23]
	Adult emergency (≥15 years)								91	21	9	2	23	WHO 1997	[23]
2005–2010	Adult emergency (≥14 years)								383	279	53			WHO 2009	[24]
2006	Territory	>53		2	0.11**		62%			21%	17%			INVS 1998	[3]
2007–2008	Territory	352	1.9–2.0	4	0.013*–0.022**	1.2*[a]				219				WHO 1997	[7,8,17,18,22,24]
2010	Territory	635–672**	1.6*	17–18	0.042**–0.045*	0.2*[b]				75				WHO 2009	[8,22]
**Guadeloupe**															
2005–2006	Territory	82	0.70*	1	0.009**	0.40*[a]	31			24	15			InVS 1998	[3,7,8,10,17,21,25]
2006	Hospitalized	31													[21]
2006–2007	Territory			2			47.0%			47.2%	5.8%				[3]
2007	Territory	272	1.40*	3	0.02**	0.80–1.0*[a]	0			159				WHO 1997	[7,8,10,17,25]
2009	District (Fond Budan, Bai Mahault)	2	11.1**			11.1**				2				WHO 1997	[26]
2009–2010	Territory	411–418	0.90	5–6	0.014**	0.30–0.36*[a]	251			156–160				WHO 1997	[8,17,22]
**Saint Martin**															
2003–2004	Territory	12–17	6.80**–7.6**	1	0.44**										[27,28]
2007–2008	Territory	22	1.03**												[29]
2008–2009	Territory					0.5[e]				4	4	2			[30]
2009–2010	Territory	20	1.11**	1		0.75[e]		5		6	4	5			[30]
**Saint Barthélemy**															

Year	Population	Hospitalizations		Deaths (n)	Case fatality rate (%)	Severity rate (%)	Non-severe cases (n)	Non-severe cases + warning signs (n)	Dengue fever (n)	Severe cases (n)	DHF (n)	DSS (n)	Others (n)	Definition used	Reference
		n	Rate (%)												
2002–2003	Territory	6	0.67												[27]
2006	Territory	147	16.5*												[21]
2006–2007	Territory	218	24.5*												[29]
2007–2008	Territory	6	1.2**			1.2**[e]				6				WHO 1997	[29,30]
2009–2010	Territory	5	1.0**			0.6**[e]				3	0	0		WHO 1997	[30]

DHF, dengue haemorrhagic fever; DSS, dengue shock syndrome; WHO, World Health Organization.

*Estimated value in the publication

**Calculated from the data available in the publication

[a]According to WHO 1997 dengue case severity classification

[b]According to WHO 2009 dengue case severity classification

[c]According to InVS 1998 dengue case severity classification

[d]According to WHO 1986 dengue case severity classification

[e]Dengue case severity classification not specified

References

1. Institut Pasteur de la Guyane (2008) Rapport annuel 2001. Available: http://www.pasteur-cayenne.fr/spip/IMG/pdf/rapport_IPG_2001.pdf Accessed: 19 November 2013
2. Mattera M, Vernerey M, Quatresous I (2006) L'épidémie de dengue survenue en Guyane en 2006. Available: http://opac.invs.sante.fr/index.php?lvl=author_see&id=7450 Accessed: 19 November 2013
3. Rosine J, Ardillon V, Cardoso T, Cassadou S, Léon L, et al. (2007) Épidémiologie de la dengue aux Antilles et en Guyane: analyse comparative des dernières épidémies, 2005-2006 et 2006-2007 [Poster]. Journées de veille sanitaire 2007, 29-30 November 2007, Paris, France.
4. Meynard J, Dussart P, Cardoso T, Langevin S, Joly N, et al. (2009) Etude de séroprévalence de la dengue chez les femmes enceintes en Guyane, 2006. Bull Epidemiol Hebd 33: 357-361.
5. National Reference Center of arboviruses and virus influenzae (2007) Rapport CNR arbovirus et virus influenza, région Antilles Guyane - Année 2006. Available: http://www.pasteur.fr/ip/resource/filecenter/document/01s-00004f-0r2/ra-cnr-arbo-ipg-2006.pdf Accessed: 19 November 2013
6. Gaisan Doncel E (2007) Analyse descriptive et comparative des 128 cas pédiatriques hospitalisés à Cayenne durant l'épidémie de dengue 2005-2006, pour lesquels ce diagnostic a été retenu [Poster]. Chikungunya et autres arboviroses émergentes en milieu tropical, 3-4 December 2007, Saint-Pierre, la Réunion.
7. Césaire R, Cabie A, Djossou F, Lamaury I, Beaucaire G, et al. (2008) Aspects récents de la dengue dans les départements français d'Amérique. Virologie 12: 151-157.
8. Quenel P, Rosine J, Cassadou S, Ardillon V, Blateau A, et al. (2011) Epidémiologie de la dengue dans les départements français d'Amérique. Bull Epidemiol Hebd 33-34: 358-363.
9. National Reference Center of arboviruses and virus influenzae (2008) Rapport CNR arbovirus et virus influenza, région Antilles Guyane - Année 2007. Available: http://www.pasteur.fr/ip/resource/filecenter/document/01s-00004f-0ps/ra-cnr-arbo-ipg-2007.pdf Accessed: 19 November 2013
10. Rosine J, Ardillon V, Cardoso T, Cassadou S, Chaud P, et al. (2007) Vers une hyperendémicité de la dengue aux Antilles-Guyane? [Poster]. Chikungunya et autres arboviroses émergentes en milieu tropical, 3-4 December 2007, Saint-Pierre, la Réunion.
11. Flamand C, Quenel P, Ardillon V, Carvalho L, Bringay S, et al. (2011) The epidemiologic surveillance of dengue fever in French Guiana: when achievements trigger higher goals. Stud Health Technol Inform 169: 629-633.
12. Djossou F, Leon L, Demar-Pierre P, Gonon S, Delattre P, et al. (2009) Pertinence des critères de sévérité observés au cours de l'épidémie de dengue DEN-2 en Guyane française en 2006 [Poster]. 10es Journées Nationales d'Infectiologie, 10-12 June 2009, Lyon, France.
13. Djossou F, Flamand C, Abboud P, Cuadro E, Hommel D, et al. (2011) Surveillance hospitalière de la dengue et comparaison de la classification OMS 2009 aux anciennes. 12es Journées Nationales d'Infectiologie, 8-10 June 2011, Toulouse, France.
14. National Reference Center of arboviruses and virus influenzae (2010) Rapport CNR arbovirus et virus influenza, région Antilles Guyane - Année 2009. Available:

http://www.pasteur.fr/ip/resource/filecenter/document/01s-00004f-0r3/ra-cnr-arbo-ipg-2009.pdf Accessed: 19 November 2013
15. National Reference Center of arboviruses and virus influenzae (2011) Rapport CNR arbovirus et virus influenza, région Antilles Guyane - Année 2010. Available: http://www.pasteur-cayenne.fr/spip/IMG/pdf/Rapport_annuel_CNRA_IPG_2010_web_vf.pdf Accessed: 19 November 2013
16. Merle S, Rosine J, Boudan V, Cicchelero V, Chaud P (2004) Estimation de l'ampleur de l'épidémie de dengue en Martinique, 2001-2002. Bull Epidemiol Hebd 45: 215-216.
17. Chappert J, Agnès M, Cassadou S, Ginhoux L, de Saint-Alary F, et al. (2011) Bilan de l'épidémie de dengue en Guadeloupe, 2010. BVS Antilles-Guyane No. 9-10 (Novembre-Décembre): 11-15.
18. Institut de Veille Sanitaire (2008) Premier bilan de l'épidémie de dengue 2007-08 en Martinique. Bulletin d'Alerte et de Surveillance Antilles Guyane 4: 10.
19. Monnin M, M'bou F (2005) An epidemic of dengue fever in a department of paediatrics: Report on 58 cases in Lamentin (Martinique). Arch Pediatr 12: 144-150.
20. Peyrefitte CN, Couissinier-Paris P, Mercier-Perennec V, Bessaud M, Martial J, et al. (2003) Genetic characterization of newly reintroduced dengue virus type 3 in Martinique (French West Indies). J Clin Microbiol 41: 5195-5198.
21. Institut de Veille Sanitaire (2006) Expérience tirée des épidémies de Martinique en 2005 et Guyane en 2006. BVS Antilles Guyane 7: 1-12.
22. Rosine J, AdélaÏde Y, Anglio J, Blateau A, Bousser V, et al. (2011) Bilan de l'épidémie de dengue en Martinique, 2010. BVS Antilles-Guyane 9-10 (Novembre-Décembre 2011): 2-6.
23. Thomas L, Verlaeten O, Cabie A, Kaidomar S, Moravie V, et al. (2008) Influence of the dengue serotype, previous dengue infection, and plasma viral load on clinical presentation and outcome during a dengue-2 and dengue-4 co-epidemic. Am J Trop Med Hyg 78: 990-998.
24. Thomas L, Moravie V, Besnier F, Valentino R, Kaidomar S, et al. (2012) Clinical presentation of dengue among patients admitted to the adult emergency department of a tertiary care hospital in Martinique: implications for triage, management, and reporting. Ann Emerg Med 59: 42-50.
25. Institut de Veille Sanitaire (2008) Premier bilan de l'épidémie de dengue 2007 en Guadeloupe. Bulletin d'Alerte et de Surveillance Antilles Guyane 4: 9.
26. Chappert J (2009) Investigation d'un foyer de dengue au quartier Fond Budan, Baie-Mahault, avril 2009. BVS Antilles-Guyane 9: 7-8.
27. Malon A, Chaud P, Gustave J (2004) Epidémie de Dengue à Saint-Martin (Guadeloupe). Rapport d'investigation. Available: http://www.invs.sante.fr/publications/2004/dengue_guadeloupe/dengue.pdf Accessed: 19 November 2013
28. Peyrefitte CN, Pastorino BA, Bessaud M, Gravier P, Tock F, et al. (2005) Dengue type 3 virus, Saint Martin, 2003-2004. Emerg Infect Dis 11: 757-761.
29. Institut de Veille Sanitaire (2008) Bilan épidémiologique de la dengue dans les îles de Saint Martin et de Saint Barthélemy, saison 2007 - 2008. Bulletin d'Alerte et de Surveillance Antilles Guyane 6: 12-13.
30. Larrieu S, Hanson S, Brin F, Chappert J, Cassadou S, et al. (2010) Bilan de la surveillance et du contrôle des épidémies de dengue à Saint-Martin et Saint-Barthélemy: saison 2009-2010. BVS Antilles-Guyane 5: 6-8.
