# Supplementary material for: Recent Epidemiological Trends of Dengue in the French Territories of the Americas (2000–2012): A Systematic Literature Review
Source: PLoS Negl Trop Dis. 2014 Nov 6;8(11):e3235. doi: 10.1371/journal.pntd.0003235 (PMC4222734; doi:10.1371/journal.pntd.0003235)
Supplement: Table S5 — Sex distribution of dengue in the French Territories of the Americas (2000–2012). (PDF) [file pntd.0003235.s005.pdf]

Table S5. Sex distribution of dengue in the French Territories of the Americas (2000–2012).

Year	Population	Sex ratio (males:females)	Reference
**French Guiana**			
2002	Territory	1.22	[1]
2005–2006	Maripasoula town	0.9	[2]
2006	Territory	1.05*	[3]
2007	Territory	0.99	[4]
2008	Territory	1.09	[5]
2008–2010	Hospitalized	0.72	[6]
2009	Territory	1.07	[7]
2010	Territory	1.16	[8]
**Martinique**			
2001–2002	Territory (0–60 years)	0.58*	[9]
	Lamentin town emergency department (0–16 years)	1.15	[10]
2005–2006	Fort-de-France emergency department (≥15 years)	0.76*	[11]
	Fort-de-France emergency department fatalities (≥15 years)	0.33*	[11]
2005–2010	Fort-de-France emergency department (≥14 years)	0.87	[12]
2010	Department fatalities (All ages)	1.12*	[13]
**Saint Martin**			
2003–2004	Baie Orientale, Cul de Sac and Mont O'Reilly districts	0.7	[14]

No data were available for Guadeloupe or Saint Barthelémy

*Calculated from the data available in the publication

References

1. Institut Pasteur de la Guyane (2008) Rapport annuel 2001. Available: http://www.pasteur-cayenne.fr/spip/IMG/pdf/rapport_IPG_2001.pdf Accessed: 19 November 2013
2. Meynard JB, Ardillon V, Venturin C, Ravachol F, Basurko C, et al. (2009) First description of a dengue fever outbreak in the interior of French Guiana, February 2006. Eur J Public Health 19: 183-188.
3. National Reference Center of arboviruses and virus influenzae (2007) Rapport CNR arbovirus et virus influenza, région Antilles Guyane - Année 2006. Available: http://www.pasteur.fr/ip/resource/filecenter/document/01s-00004f-0r2/ra-cnr-arbo-ipg-2006.pdf Accessed: 19 November 2013
4. National Reference Center of arboviruses and virus influenzae (2008) Rapport CNR arbovirus et virus influenza, région Antilles Guyane - Année 2007. Available: http://www.pasteur.fr/ip/resource/filecenter/document/01s-00004f-0ps/ra-cnr-arbo-ipg-2007.pdf Accessed: 19 November 2013
5. National Reference Center of arboviruses and virus influenzae (2009) Rapport CNR arbovirus et virus influenza, région Antilles Guyane - Année 2008. Available: http://www.pasteur.fr/ip/resource/filecenter/document/01s-00004f-0pt/ra-cnr-arbo-ipg-2008.pdf Accessed: 19 November 2013
6. Djossou F, Flamand C, Abboud P, Cuadro E, Hommel D, et al. (2011) Surveillance hospitalière de la dengue et comparaison de la classification OMS 2009 aux anciennes. 12es Journées Nationales d'Infectiologie, 8-10 June 2011, Toulouse, France.
7. National Reference Center of arboviruses and virus influenzae (2010) Rapport CNR arbovirus et virus influenza, région Antilles Guyane - Année 2009. Available: http://www.pasteur.fr/ip/resource/filecenter/document/01s-00004f-0r3/ra-cnr-arbo-ipg-2009.pdf Accessed: 19 November 2013
8. National Reference Center of arboviruses and virus influenzae (2011) Rapport CNR arbovirus et virus influenza, région Antilles Guyane - Année 2010. Available: http://www.pasteur-cayenne.fr/spip/IMG/pdf/Rapport_annuel_CNRA_IPG_2010_web_vf.pdf Accessed: 19 November 2013
9. Merle S, Rosine J, Boudan V, Cicchelero V, Chaud P (2004) Estimation de l'ampleur de l'épidémie de dengue en Martinique, 2001-2002. Bull Epidemiol Hebd 45: 215-216.
10. Monnin M, M'bou F (2005) An epidemic of dengue fever in a department of paediatrics: Report on 58 cases in Lamentin (Martinique). Arch Pediatr 12: 144-150.
11. Thomas L, Verlaeten O, Cabie A, Kaidomar S, Moravie V, et al. (2008) Influence of the dengue serotype, previous dengue infection, and plasma viral load on clinical presentation and outcome during a dengue-2 and dengue-4 co-epidemic. Am J Trop Med Hyg 78: 990-998.
12. Thomas L, Moravie V, Besnier F, Valentino R, Kaidomar S, et al. (2012) Clinical presentation of dengue among patients admitted to the adult emergency department of a tertiary care hospital in Martinique: implications for triage, management, and reporting. Ann Emerg Med 59: 42-50.
13. Rosine J, AdélaÏde Y, Anglio J, Blateau A, Bousser V, et al. (2011) Bilan de l'épidémie de dengue en Martinique, 2010. BVS Antilles-Guyane 9-10 (Novembre-Décembre 2011): 2-6.
14. Malon A, Chaud P, Gustave J (2004) Epidémie de Dengue à Saint-Martin (Guadeloupe). Rapport d'investigation. Available:

http://www.invs.sante.fr/publications/2004/dengue_guadeloupe/dengue.pdf Accessed: 19 November 2013
